# Correction: Cover crops support the climate change mitigation potential of agroecosystems

**DOI:** 10.1371/journal.pone.0319516

**Published:** 2025-02-12

**Authors:** Jonas Schön, Norman Gentsch, Peter Breunig

Figs 2 and 3, Table 2 and S1 and S2 Files are incorrect. The results of the studies Engedal et al. 2023 and Notoris et al. 2019 should have not been included in the quantification carbon sequestration effects of cover crops. Please see the correct Figs 2 and 3, Table 2 and S1 and S2 Files here.

**Fig 2:**
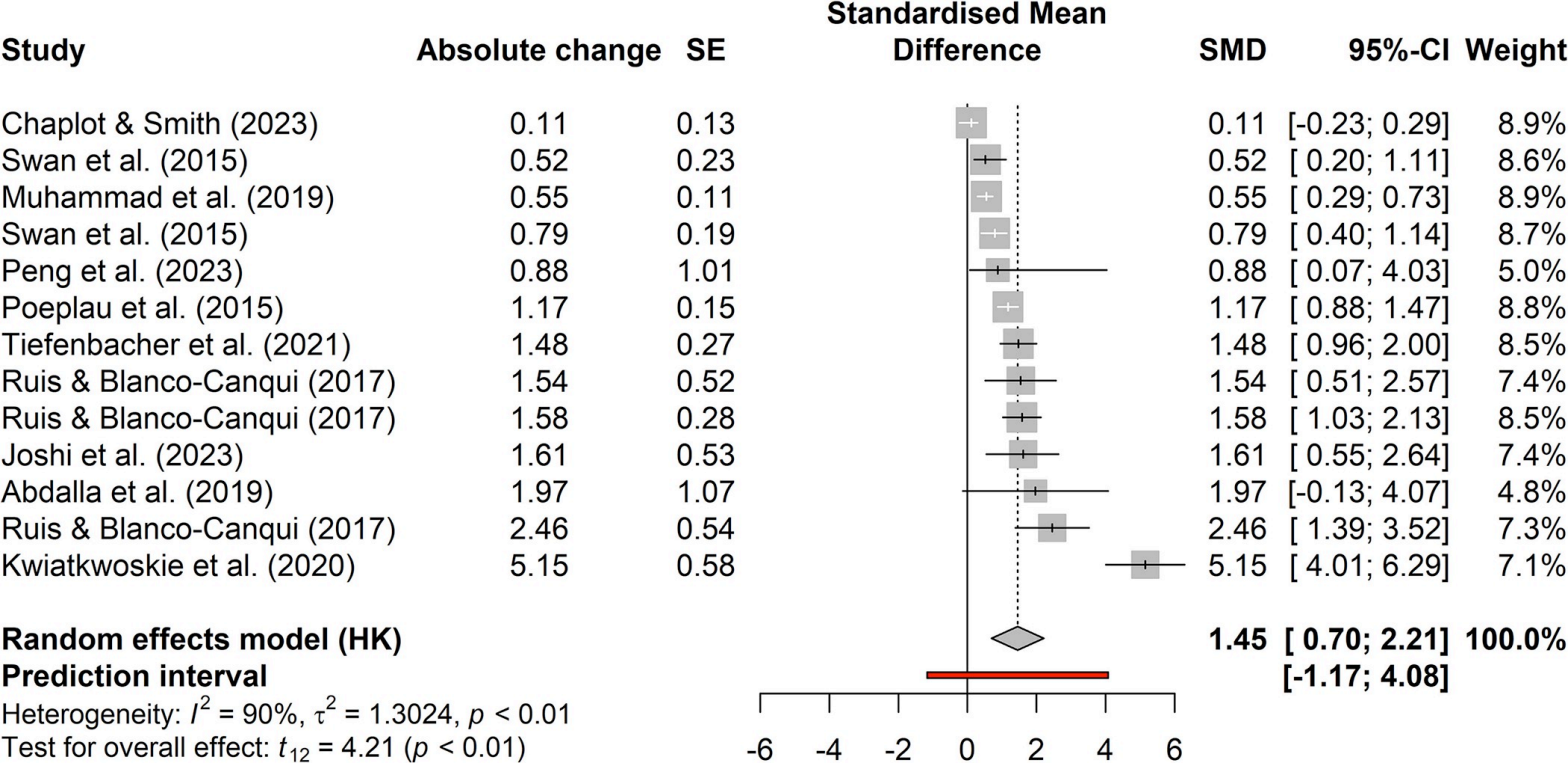
Forest plot of included literature on carbon sequestration effects.

**Fig 3:**
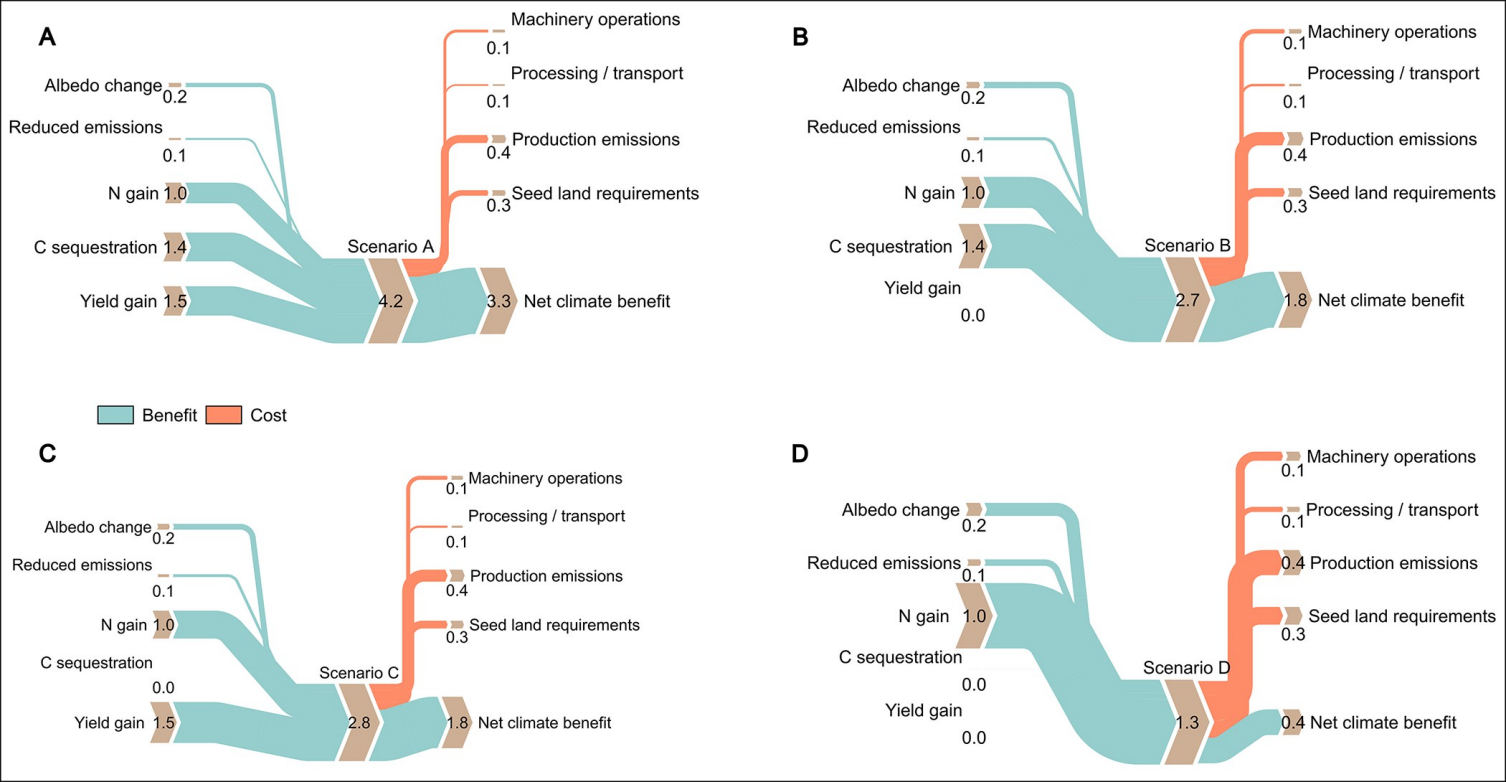
Sankey plots describing gains and losses of different scenarios for the NCCMI.

**Table 2:** Calculation of the net climate change mitigation impact of cover crops during an annual cropping cycle.

	EU27 Average per ha				EU27 total			
		Mean	Low (-95% Conf. Level)	High (+95% Conf. Level)		Mean	Low (-95% Conf. Level)	High (+95% Conf. Level)
Area	ha				1.000 ha	15,092.14	14,785.60	15,398.67
Average Yield	Mg/ha	7.88	7.46	8.31				
Relative Yield Gain	%	8.8%	2.9%	14.8%				
Absolute Yield Gain	Mg/ha	0.70	0.21	1.23				
COC Maize Grains	kg CO_2_e/kg	2.10	2.10	2.10				
Carbon Land Benefit based on Yield Gain	Mg CO_2_e/ha	1.46	0.45	2.59	1.000 Mg CO_2_e	22,086.83	6,631.87	39,805.69
Carbon Sequestration	Mg CO_2_/ha	1.43	0.89	1.97	1.000 Mg CO_2_e	21,608.24	13,170.77	30,377.36
Nitrogen Fixation	kg N/ha	50.15	29.72	70.58				
Reduced Nitrogen Leaching	kg N/ha	38.82	19.13	58.51				
Total Nitrogen Fertilizer Savings	kg N/ha	88.98	48.85	129.10				
Nitrogen Fertilizer Emissions	kg CO_2_e/kg N	11.23	9.02	13.43				
Carbon Benefit based on Nitrogen Fertilizer Savings	Mg CO_2_e/ha	1.00	0.44	1.73	1.000 Mg CO_2_e	15,077.55	6,518.18	26,703.02
Reduced N2O Emissions due to less Leaching	Mg CO_2_e/ha	0.10	-0.15	0.35	1.000 Mg CO_2_e	1,495.60	-2,218.43	5,362.37
Albedo change	Mg CO_2_e/ha	0.20	-0.37	0.78	1.000 Mg CO_2_e	3,087.10	-5,514.25	12,042.49
Total Climate Benefit	Mg CO_2_e/ha	4.20	1.26	7.42	1.000 Mg CO_2_e	63,355.31	18,588.15	114,290.92
N2O Emissions	Mg CO_2_e/ha	0.04	-0.02	0.09	1.000 Mg CO_2_e	555.77	-306.57	1,453.41
Land Requirements vor Catch Crop Seed Production								
Mustard	ha Seed/ha CC	0.01						
Oats	ha Seed/ha CC	0.02						
Phacelia	ha Seed/ha CC	0.03						
Clover	ha Seed/ha CC	0.04						
Average	ha Seed/ha CC	0.02	0.02	0.02				
COC Wheat	kg CO_2_e/kg	1.90	1.90	1.90				
Average Yield Soft Wheat	Mg/ha	5.72	5.54	5.90				
Carbon Benefit Wheat	Mg CO_2_e/ha	10.86	10.52	11.20				
Net Avoided Production Emissions Wheat	Mg CO_2_e/ha	0.29	0.29	0.29				
Foregone Net GHG Benefit of Wheat due to CC Seed Land Requirements	Mg CO_2_e/ha	0.28	0.27	0.29	1.000 Mg CO_2_e	4,180.56	3,970.76	4,395.55
Production Emissions Catch Crop Seed	Mg CO_2_e/ha	0.39	0.08	0.70	1.000 Mg CO_2_e	5,920.55	1,194.38	10,837.69
Processing, Packaging and Transport of Catch Crop Seed	Mg CO_2_e/ha	0.08	0.08	0.08	1.000 Mg CO_2_e	1,182.71	1,158.69	1,206.74
Additional Machinery Operations	l Diesel/ha	45.29	32.44	58.13				
Diesel Emissions	kg CO_2_/l	3.13	3.13	3.13				
Additonal Machinery Operations	Mg CO_2_/ha	0.14	0.10	0.18	1.000 Mg CO_2_e	2,140.93	1,502.51	2,804.02
Total Climate Cost	Mg CO_2_e/ha	0.93	0.51	1.34	1.000 Mg CO_2_e	13,980.53	7,519.77	20,697.40
Net Climate Change Mitigation Impact of Cover Crops	Mg CO_2_e/ha	3.27	0.75	6.08	1.000 Mg CO2e	49,374.79	11,068.38	93,593.52
Share of EU- 27 agricultural emissions in 2020						12.9%	2.9%	24.5%

In the Abstract section, there is an error in the fourth and tenth sentences. The correct fourth sentence is: The results indicate that cover crops lead to a net climate change mitigation impact (NCCMI) of 3.27 Mg CO_2_e ha^-1^ a^-1^. The correct tenth sentence is: Extrapolating these results, planting cover crops before all maize acreage in the EU results in a climate change mitigation of 49.37 million Mg CO_2_e a^-1^, which is equivalent to 12.9% of the EU’s agricultural emissions.

In the Methods section, there is an error in the third sentence of the fourth paragraph. The correct sentence is: Only 41 articles met these criteria and were finally included in the review. In addition, we used 31 publications from Google Scholar and data from 26 other Internet sources that were selected based on the above criteria.

In the Carbon sequestration subsection of Results, there is an error in the third and eighth sentences of the first paragraph. The correct third sentence is: In addition, 24 studies that were not included in the above-mentioned meta-analyses were also included in our calculations. The correct eighth sentence is: The weighted mean of all studies investigated shows an average sequestration of 1.43 Mg CO_2_e ha^-1^ a^-1^ (95% CI, 0.89, 1.97).

In Nitrogen fixation subsection of Results, there is an error in the fourth, fifth and eighth sentences of the paragraph. The fourth and fifth sentence should have been a single sentence and written as: For example, winter vetch, which fixes 85.00 kg N ha^-1^ as single species and 113.00 kg N ha^-1^ in a mixture with winter rye [13]. The correct eighth sentence is: The summarized literature resulted in a weighted mean of 50.15 kg N ha-1 (95% CI, 29.72, 70.58) that can be contributed by legumes from nitrogen fixation.

The paragraph in Net climate change mitigation impact (NCCMI) of cover crops subsection of Results is incorrect. The correct paragraph is: We calculate the summary of all climate benefits from cover crops as 4.20 Mg CO_2_e ha^-1^ a^-1^ (95%CI, 1.26, 7.42) and all climate costs from cover crops as 0.93 Mg CO_2_-eq ha^-1^ a^-1^ (95% CI 0.51, 1.34). The NCCMI of cover crops was calculated as 3.27 Mg CO_2_e ha^-1^ a^-1^ (Table 3). The NCCMI was then extrapolated to the maize harvest area for all EU-27 countries. Here, we assume a scenario in which cover crops are grown before maize on all of the EU-27’s harvested maize areas, which is 15,092 x 10^6^ ha, on average [23]. Based on this calculation, planting cover crops before maize in the EU-27 results in a climate change mitigation potential of 49.37 million Mg CO_2_e a^-1^. This is equivalent to 12.9% of the EU-27’s agricultural GHG emissions [51].

In Review of results subsection of Discussion, there is an error in the second and 14^th^ sentences. The correct second sentence is: This resulted in a positive NCCMI of 3.27 Mg CO_2_e ha^-1^ a^-1^ that can be achieved if cover crops were incorporated in crop rotations of maize production. The correct 14^th^ sentence is: Closer nutrient cycling and reduction of leaching losses resulted in another 23.8% of cover crops’ climate benefits, including nitrogen fixation of legume cover crops.

In the Conclusion and policy recommendations subsection of Discussion, there is an error in the third sentence of the first paragraph. The correct sentence is: If all maize cropping acreage in the EU-27 were to include cover crops, GHG emissions from EU-27 agriculture could be reduced by 12.9%.

Notoris et al. was incorrectly included as reference 27. As a result, all subsequent references are misnumbered. References 28–66 should be references 27–65.

## Supporting information

S1 File Calculations. (XLSX)

S2 File Included literature. (XLSX)
