# Supplementary material for: Cover crops support the climate change mitigation potential of agroecosystems
Source: PLoS One. 2024 May 8;19(5):e0302139. doi: 10.1371/journal.pone.0302139 (PMC11078372; doi:10.1371/journal.pone.0302139)
Supplement: Attachment — Submitted filename: PONE-D-23-39906_reviewer.pdf [file pone.0302139.s003.pdf]

PLOS ONE

Cover crops support the climate change mitigation potential of agroecosystems

--Manuscript Draft--

**Manuscript Number:**	PONE-D-23-39906
**Article Type:**	Research Article
**Full Title:**	Cover crops support the climate change mitigation potential of agroecosystems
**Short Title:**	Cover crops support the climate change mitigation potential of agroecosystems
**Corresponding Author:**	Peter Breunig<br>Hochschule Weihenstephan-Triesdorf<br>Weidenbach, GERMANY
**Keywords:**	cover crops; climate change mitigation; carbon opportunity cost; carbon sequestration; maize
**Abstract:**	Cover crops have the potential to mitigate climate change by reducing negative impacts of agriculture on ecosystems. This study is first to quantify the net climate change mitigation impact of cover crops including land-use effects. A systematic literature review was conducted to identify major drivers for climate benefits and costs of cover crops in maize (Zea maize L.) production systems. The results indicate that cover crops lead to a net climate change mitigation impact of 3.30 Mg CO2e ha-1 a-1, of which carbon land benefits based on maize yield gains following cover crops is the major contributor (34.5% of all benefits). Carbon sequestration is the second largest contributor (33.8%). The climate costs of cover crops are mainly dominated by emissions from their seed production and foregone benefits due to land use for cover crops seeds. However, these two costs account for only 15.8% of the benefits. Extrapolating these results, planting cover crops before all maize acreage in the EU results in a climate change mitigation of 49.80 Mio. Mg CO2e a-1, which is equivalent to 13.0% of the EU's agricultural emissions. This study highlights the importance of incorporating cover crops into sustainable cropping systems to mitigate climate change.
**Order of Authors:**	Jonas Schön
	Norman Gentsch
	Peter Breunig
**Additional Information:**	
**Question**	**Response**
**Financial Disclosure**<br><br>Enter a financial disclosure statement that describes the sources of funding for the work included in this submission. Review the submission guidelines for detailed requirements. View published research articles from *PLOS ONE* for specific examples.<br><br>This statement is required for submission and **will appear in the published article** if the submission is accepted. Please make sure it is accurate.	Yes

Powered by Editorial Manager® and ProduXion Manager® from Aries Systems Corporation

**Funded studies**<br>Enter a statement with the following details:<br>• Initials of the authors who received each award<br>• Grant numbers awarded to each author<br>• The full name of each funder<br>• URL of each funder website<br>• Did the sponsors or funders play any role in the study design, data collection and analysis, decision to publish, or preparation of the manuscript?<br><br>Did you receive funding for this work?	
Please add funding details.<br>as follow-up to "**Financial Disclosure**<br><br>Enter a financial disclosure statement that describes the sources of funding for the work included in this submission. Review the submission guidelines for detailed requirements. View published research articles from *PLOS ONE* for specific examples.<br><br>This statement is required for submission and **will appear in the published article** if the submission is accepted. Please make sure it is accurate.<br><br>**Funded studies**<br>Enter a statement with the following details:<br>• Initials of the authors who received each award<br>• Grant numbers awarded to each author<br>• The full name of each funder<br>• URL of each funder website<br>• Did the sponsors or funders play any role in the study design, data collection and analysis, decision to publish, or preparation of the manuscript?<br><br>Did you receive funding for this work?"	The research was funded by the German Federal Ministry of Education and Research within the Project "CATCHY", project number: 031B1060C
Please select the country of your main research funder (please select carefully as in some cases this is used in fee calculation).	GERMANY - DE

as follow-up to "**Financial Disclosure**<br><br>Enter a financial disclosure statement that describes the sources of funding for the work included in this submission. Review the submission guidelines for detailed requirements. View published research articles from *PLOS ONE* for specific examples.<br><br>This statement is required for submission and **will appear in the published article** if the submission is accepted. Please make sure it is accurate.<br><br>**Funded studies**<br>Enter a statement with the following details:<br>• Initials of the authors who received each award<br>• Grant numbers awarded to each author<br>• The full name of each funder<br>• URL of each funder website<br>• Did the sponsors or funders play any role in the study design, data collection and analysis, decision to publish, or preparation of the manuscript?<br><br>Did you receive funding for this work?"	
**Competing Interests**<br><br>Use the instructions below to enter a competing interest statement for this submission. On behalf of all authors, disclose any competing interests that could be perceived to bias this work—acknowledging all financial support and any other relevant financial or non-financial competing interests.<br><br>This statement is required for submission and **will appear in the published article** if the submission is accepted. Please make sure it is accurate and that any funding sources listed in your Funding Information later in the submission form are also declared in your Financial Disclosure statement.	The authors have declared that no competing interests exist.

View published research articles from *PLOS ONE* for specific examples.<br><br>**NO authors have competing interests**<br><br>Enter: *The authors have declared that no competing interests exist.*<br><br>**Authors with competing interests**<br><br>Enter competing interest details beginning with this statement:<br><br>*I have read the journal's policy and the authors of this manuscript have the following competing interests: [insert competing interests here]*<br><br>* typeset	
**Ethics Statement**<br><br>Enter an ethics statement for this submission. This statement is required if the study involved:<br><br>• Human participants<br>• Human specimens or tissue<br>• Vertebrate animals or cephalopods<br>• Vertebrate embryos or tissues<br>• Field research<br><br>Write "N/A" if the submission does not require an ethics statement.<br><br>General guidance is provided below. Consult the submission guidelines for detailed instructions. **Make sure that all information entered here is included in the Methods section of the manuscript.**	N/A

Powered by Editorial Manager® and ProduXion Manager® from Aries Systems Corporation

**Format for specific study types**<br><br>**Human Subject Research (involving human participants and/or tissue)**<br>• Give the name of the institutional review board or ethics committee that approved the study<br>• Include the approval number and/or a statement indicating approval of this research<br>• Indicate the form of consent obtained (written/oral) or the reason that consent was not obtained (e.g. the data were analyzed anonymously)<br><br>**Animal Research (involving vertebrate animals, embryos or tissues)**<br>• Provide the name of the Institutional Animal Care and Use Committee (IACUC) or other relevant ethics board that reviewed the study protocol, and indicate whether they approved this research or granted a formal waiver of ethical approval<br>• Include an approval number if one was obtained<br>• If the study involved *non-human primates*, add *additional details* about animal welfare and steps taken to ameliorate suffering<br>• If anesthesia, euthanasia, or any kind of animal sacrifice is part of the study, include briefly which substances and/or methods were applied<br><br>**Field Research**<br><br>Include the following details if this study involves the collection of plant, animal, or other materials from a natural setting:<br>• Field permit number<br>• Name of the institution or relevant body that granted permission	
**Data Availability**<br><br>Authors are required to make all data underlying the findings described fully available, without restriction, and from the time of publication. PLOS allows rare exceptions to address legal and ethical concerns. See the PLOS Data Policy and FAQ for detailed information.	Yes - all data are fully available without restriction

A Data Availability Statement describing where the data can be found is required at submission. Your answers to this question constitute the Data Availability Statement and **will be published in the article**, if accepted.<br><br>**Important:** Stating 'data available on request from the author' is not sufficient. If your data are only available upon request, select 'No' for the first question and explain your exceptional situation in the text box.<br><br>Do the authors confirm that all data underlying the findings described in their manuscript are fully available without restriction?	
**Describe where the data may be found in full sentences. If you are copying our sample text, replace any instances of XXX with the appropriate details.**<br><br>• If the data are **held or will be held in a public repository**, include URLs, accession numbers or DOIs. If this information will only be available after acceptance, indicate this by ticking the box below. For example: *All XXX files are available from the XXX database (accession number(s) XXX, XXX.*).<br>• If the data are all contained **within the manuscript and/or Supporting Information files**, enter the following: *All relevant data are within the manuscript and its Supporting Information files.*<br>• If neither of these applies but you are able to provide **details of access elsewhere**, with or without limitations, please do so. For example:<br><br>*Data cannot be shared publicly because of [XXX]. Data are available from the XXX Institutional Data Access / Ethics Committee (contact via XXX) for researchers who meet the criteria for access to confidential data.*<br><br>*The data underlying the results presented in the study are available from (include the name of the third party*	All relevant data are within the manuscript and its Supporting Information files.

and contact information or URL).<br>• This text is appropriate if the data are owned by a third party and authors do not have permission to share the data.<br><br>* typeset	
Additional data availability information:	

Manuscript Click here to access/download;Manuscript;Manuscript.docx

Cover crops support the climate change mitigation potential of agroecosystems

Jonas Schön[1], Norman Gentsch[2], Peter Breunig[3]

[1]Weihenstephan-Triesdorf University of Applied Sciences, Triesdorf, Germany, jonas.schoen@hswt.de, ORCID: 0009-0007-6550-9616

[2]Institute of Soil Science, Leibniz Universität Hannover, Hannover, 30419, Germany, gentsch@ifbk.uni-hannover.de, ORCID: 0000-0003-1166-8973

[3]Weihenstephan-Triesdorf University of Applied Sciences, Triesdorf, Germany, peter.breunig@hswt.de, ORCID: 0000-0003-3661-7830

Abstract

Cover crops have the potential to mitigate climate change by reducing negative impacts of agriculture on ecosystems. This study is first to quantify the net climate change mitigation impact of cover crops including land-use effects. A systematic literature review was conducted to identify major drivers for climate benefits and costs of cover crops in maize (*Zea maize L.*) production systems. The results indicate that cover crops lead to a net climate change mitigation impact of 3.30 Mg CO_2e ha^{-1} a^{-1}, of which carbon land benefits based on maize yield gains following cover crops is the major contributor (34.5% of all benefits). Carbon sequestration is the second largest contributor (33.8%). The climate costs of cover crops are mainly dominated by emissions from their seed production and foregone benefits due to land use for cover crops seeds. However, these two costs account for only 15.8% of the benefits.

Extrapolating these results, planting cover crops before all maize acreage in the EU results in a climate change mitigation of 49.80 Mio. Mg CO_2e a^{-1}, which is equivalent to 13.0% of the EU’s agricultural emissions. This study highlights the importance of incorporating cover crops into sustainable cropping systems to mitigate climate change.

Keywords: cover crops, climate change mitigation, carbon opportunity cost, carbon sequestration, maize

1. Introduction

Mitigating climate change through measures in agriculture and food systems is essential for achieving the 1.50° and 2.00°C climate change targets of the Paris Agreement[1]. Worldwide, agriculture and food production systems are responsible for about one-third of global anthropogenic greenhouse gas (GHG) emissions, of which 40.0% are caused by agricultural production and 32.0% by land use and land use change[2]. However, agricultural systems also offer opportunities for climate change mitigation. Agroecosystems have significant potential to reduce global net emissions[3] and could even act as a net carbon sink[4]. To achieve these emission reduction and carbon sequestration opportunities, a transformation of agricultural systems is required. An essential component of climate-smart agricultural systems is planting cover crops. Cover crops are plants or plant mixtures that are not harvested for revenue, but are grown for the purpose of soil nutrient management, organic matter input, soil protection, and soil health improvement[5]. Cover crops are associated with multiple ecosystem services, such as closer nutrient cycling[5,6], activation of soil biology, biodiversity improvement[7], soil water management, and restoration of soil structures[8]. In addition, the mentioned effects of cover crops could mitigate climate change by soil carbon sequestration[9], biomass carbon storage, or reduced fertilizer losses to aquatic ecosystems. Recent studies show that these

effects can lead to agronomic benefits that translate into higher yields as well as lower required fertilizers, pesticides, and water input[10].

Despite these benefits, there exists a knowledge gap. In the scientific literature thus far, there are no complete climate impact assessment of the cultivation of cover crops that also fully include land use effects. Cover crops reduces land use requirements by increasing yields of the subsequent crop. As land use change is still the largest carbon emissions source in the agri-food-system, yield increasing measures can be considered as a climate change mitigation option. According to Searchinger et al. [11], these carbon land benefits can be calculated as a "carbon opportunity cost," which is the foregone carbon storage potential from natural vegetation to produce a certain agricultural product in kg CO_2e kg^{-1}. When yields rise on one piece of land, this carbon storage potential can be maintained or restored on other locations since land use change is prevented or reverted. Kovak et al. [12] used this approach to quantify the climate change mitigation potential of a yield increase based on genetic modified crops. This is the first study to include carbon opportunity cost in the evaluation of the climate impact of cover cropping. As an additional climate benefit, cover crops remove carbon from the atmosphere during their growth while their residues and rhizodeposits are transformed to stable soil organic matter. Further climate benefits of cover crops can be attributed to the supply of nutrients to the subsequent crop, which allows for fertilizer savings[13], reduced N_2O emissions due to less nitrogen leaching, reduced solar radiation due to plant growth[14], and reduced soil erosion[15]. We define all factors above as "climate benefits".

While cultivating cover crops can accrue these climate benefits, it can also generate GHG emissions that we refer as "climate costs". The cultivation of cover crops can increase N_2O emissions in periods of anaerobic microbial conversion of cover crop decomposition products[16]. Emissions are also generated in the production of cover crop seeds through a

number of avenues, such as the land required for seed production and the production process of the seeds. In addition, produced seeds need to be packed and delivered, which also generates emissions[17]. Lastly, tilling, seeding, and termination of cover crops requires the use of GHG emitting machinery and resources[18]. Both climate benefits and climate costs of cover crops need to be carefully considered to calculate the NCCMI.

This research is first to develop a comprehensive framework to quantify the NCCMI of cover crops including land use effects based on a systematic literature review. Using selected keywords, a systematic search was conducted in “Web of Science” and “Google Scholar” and resulted in 1269 publications. We define a list of relevant climate benefits and climate costs from which the NCCMI of cover crops is derived. We apply the framework to cover crops for maize in the European Union (EU-27, which is all EU Member States excluding the United Kingdom) to quantify its total NCCMI.

2. Methods

The following analyses are based on a typical Central European crop rotation, namely winter cover crops that are incorporated into the soil and maize as a cash crop following the cover crops in the spring. To quantify the impact of cover crop cultivation on the climate mitigation potential, a systematic literature review was conducted. The research platforms "Web of Science" and "Google Scholar" were used to explore the literature. Based on the initial research, we defined five factors that support climate change mitigation (“climate benefits”) and five factors leading to additional GHG emissions (“climate costs”) due to cover cropping (Table). The individual research terms of the individual impact factors are described below in detail.

Table 1: Impact factors of cover crops on climate change mitigation analyzed in this study

**Climate Benefits**	**Climate Costs**
- Carbon land benefit based on yield gain	- N_2O emissions
- Carbon sequestration	- Foregone benefits due to cover crop seed land use
- Nitrogen fertilizer savings	- Production emissions for cover crop seed production
- Reduced indirect N_2O emissions due to less leaching	- Processing, packaging, and transport of cover crop seed
- Albedo change	- Additional machinery operations

To calculate the NCCMI of cover crops, the values of all impact factors were converted to the same unit, namely megagram of CO_2 equivalents per hectare and year (Mg CO_2e ha^{-1} a^{-1}). From the values in the literature, a weighted average value that takes into account the number of measurements in the specific study was calculated. Only data from the literature that indicated the number of measurement points were considered in the calculation of the NCCMI. Thus, the climate benefits and climate costs could be added together in each case before subtracting the costs from the benefits. This resulted in a value for the NCCMI per hectare of cover crops given in Mg CO_2e ha^{-1} a^{-1}. The 95% confidence interval was calculated from all study means and presents a range of estimates. To extrapolate the results to the EU-27 maize area the respective impact factors and the climate balances were subsequently multiplied with the maize acreage in the EU-27. The result was then divided by the total emissions from agriculture in the EU-27. This calculation resulted in the share of the NCCMI of cover crops before maize relative to all EU-27 agricultural emissions. The average maize acreage in 22 of the EU-27 countries was calculated as an average based on the years 2011 to 2022 (five of the EU-27 countries do not grow relevant quantities of maize and were thus not considered.)

The following search terms were used in Web of Science:

Cover crop & albedo, Cover crop & C sequestration, Cover crop & Yield corn, Cover crop & Yield maize, Cover crop & life cycle assessment, Cover crop & N-fixation, Cover crop & N-leaching, Fertilizer & Greenhouse gas emissions, Greenhouse gas emissions & fertilizer production, Cover crop & N_2O, Catch crop & N_2O. To enable a phrase search, all search terms were used with quotation marks to obtain search results exactly in this word order.

A total of 883 publications were extracted and checked for their suitability. Criteria for the required data were an absolute value from the respective study and the number of measurements. Only 44 articles met these criteria and were finally included in the review. In addition, we used 31 publications from Google Scholar and data from 26 other Internet sources that were selected based on the above criteria. In selecting sources, cover crops were considered both consisting of individual components and mixtures, overwintering as well as freezing, and legumes as well as non-legumes. All data sources are presented in the supplemental material (Excel file S1) and sorted based on the subsections below.

2.1. Carbon land benefit based on yield gain

The yield gain in maize following cover crops was calculated by multiplying the relative yield increase from the literature with the average maize yield in the EU-27. For this purpose, the harvest volume of silage and grain maize in Europe was divided by the cultivated area. Since carbon opportunity costs factors published in Searchinger et al.[11] refer only to grain maize, the silage maize (SM) yields were transformed to grain maize (GM). It was assumed that 54% of the dry matter of silage maize is grain [19]. Thus, the fresh mass of silage corn was multiplied by a factor of 0.35 to calculate the dry matter and then multiplied by 0.54[19] to generate the grain yield of SM (see the supplemental material for more detail). To obtain the grain yield

based on the standard of 14% moisture, the result was divided by 0.86. The following formula shows the complete calculation to transfer SM to GM:

$$GM = \frac{SM \times 0.35 \times 0.54}{0.86}.$$

To quantify the effect of yield increases on climate change mitigation, we use the carbon land benefit approach of Searchinger et al.[11]. This approach quantifies the benefit of increasing yields on one piece of land through the carbon storage potential of native vegetation on other land that is not required to produce a certain quantity of product because of higher production on existing agricultural land. According to Searchinger et al.[11], the carbon land benefit carbon opportunity costs (COC_s) is defined as follows:

$$COC_s = Y \times CO,$$

whe[illegible]OC$_s$ is the carbon land benefit, Y is the grain and biomass yield of an agricultural product, and CO[illegible] the carbon opportunity cost factor for the agricultural product. COC quantifies the forgone carbon storage potential of the native vegetation of land, which is globally used to produce an agricultural commodity and given in kg CO_2e per kg of product. Searchinger et al.[11] use two methods to quantify the carbon opportunity cost. Here, we use the values of the so-called “loss-method.” To quantify the carbon land benefit caused by yield gains in maize through cover crops, the absolute yield increase of maize in kg ha^{-1} is multiplied by 2.1 kg CO_2e kg^{-1}, which is the carbon opportunity cost factor of grain maize published in Searchinger et al.[11].

2.2. Carbon sequestration

Carbon sequestration through cover crops is usually provided in units of soil organic carbon. This value was multiplied by a factor of 3.667 (i.e., the ratio of molar masses of CO2 [illegible]d C) to obtain the value of CO_2 stored per ha land.

2.3. Nitrogen fertilizer savings

For nutrient savings, we considered only the main nutrient, namely nitrogen since its production is highly energy intensive. To quantify fertilizer savings, we only considered emissions of mineral fertilizers and not that of organic fertilizers. The overall nitrogen fertilizer savings were quantified as the sum of the mean literature values on nitrogen scavenging and the mean literature values of nitrogen fixation from cover crops.

2.4. Reduced indirect N_2O emissions due to less leaching

Reducing nitrogen leaching due to cover crops leads to lower indirect N_2O emissions since less nitrogen is deposited to rivers and other water bodies. To quantify this effect, we use the approach suggested in the 2006 IPCC Guidelines for National Greenhouse Gas Inventories: The amount of nitrogen leaching reduction is multiplied by the emission factor EF_5 of 0.0075 [20] to obtain the reduction in indirect N_2O emissions per hectare. This value is then converted from kg N_2O-N ha^{-1} to kg CO_2 -eq ha^{-1} using the conversion factor 273 and divided by 1000 to obtain the unit Mg CO_2 -eq ha^{-1}.

$$Reduced\ N_2O - N = \frac{EF_5 * Reduced\ N_{Leaching} * 273}{1000}$$

2.5 Albedo change

The reduced adsorption and thus increased reflection of solar radiation by cover crops' plant cover compared to fallow land leads to a climate change mitigation effect. We found, however, only two studies evaluating the impact of albedo change from cover crops. One by Kaye and Quemada [21] and another by Carrer et al. [14].

2.6. Nitrous oxide emissions

In the case of nitrous oxide emissions, the cumulative emissions that occur during the period of fallow or the period from sowing to incorporation of cover crops are considered. The values with the unit kg N_2O-N ha^{-1} were multiplied by a factor of 273 and 1000 to obtain the unit in Mg CO_2e ha^{-1}.

2.7. Foregone benefits due to cover crop seed land use

The production of seeds for cover crops requires additional land that is not available for production of agricultural products. Land for these seeds cannot create the same carbon benefits[11] as alternative uses of this land. Therefore, this forgone carbon benefit needs to be added to the climate costs of cover crops. For this purpose, the yields of the four most common seed components of a cover crop mixture were determined in order to calculate the average land requirement for seed production of one hectare of cover crops' seed mixture based on typical seeding rates. The following four species were selected to determine the land

use for cover crops' seed production: phacelia *(Phacelia tanecetifolia Benth.)*, mustard *(Sinapis alba L.)*, clover *(Trifolium alexandrinum L.)*, and oats *(Avena strigose Schreb.)*. Each of these cover crops has a 25% share in the final mixture of cover crops. A literature search on seed yields and average seeding rates for these four species was conducted. The seed land use (SLU) for a specific cover crop was calculated per ha based on the following formula:

$$SLU = \frac{1}{n}\sum_{i=1}^{n}\left(\frac{Y_{cc}}{SR_{cc}}\right)i,$$

where Y_{cc} is the yield in kg ha^{-1} of a specific cover crop and SR_{cc} is the seeding ratio in kg ha^{-1}. Table shows the mean input values derived from the literature for the land use requirements for cover crop seeds.

Table 2: Calculation for mean land use for cover crops' seed production

**Species**	**Seed yield in kg ha^{-1}**	**Seeding rate in kg ha^{-1}**	**Seed land use in ha seed ha^{-1} for cover crops**
Phacelia	350.00	10.50	0.03
Mustard	1,738.00	20.00	0.01
Clover	732.00	29.17	0.04
Oats	4,869.50	87.50	0.02
**Mixture**			**0.03**

The forgone carbon benefit of the land used for the production of cover crop seeds is determined as follows: We assume that seeds for cover crops are produced in the European Union on land that would otherwise be used for wheat production since wheat is the largest crop in the EU by acreage. The carbon benefit (CB) of wheat is defined by Searchinger et al.[11]. Here, we assume that the carbon stock of land is not changing between wheat and seed production for cover crops and that there are no carbon benefits from bioenergy. The equation for CB is as follows:

$$CB = (COC + PEM_{avg} - PEM_{EU}) \times Y$$

where COC is the global carbon opportunity cost factor of wheat, PEM_{avg} is the global average production emissions for wheat, PEM_{EU} is the European Union's production emissions for wheat, and Y is the average wheat yield in the European Union (based on data from 2011 to 2022 for the EU-27[22]). COC, PEM_{avg}, and PEM_{EU} are from Searchinger et al.[11]. For PEM_{EU}, we used the production emissions for wheat in Sweden, since no European average values were available. The forgone carbon benefits due to seed land use can be calculated by multiplying SLU and CB.

2.8. Production emissions for seed production, dispatch and additional machinery costs

Product emissions for seed production of cover crops are not available in literature and therefore derived from maize, rapeseed, and spring wheat seed production. Emissions data is collected from the literature in kg CO_2e kg^{-1} of seed and is multiplied with the seeding rate of cover cro[illegible]This results in an emissions value per hectare.

Emissions for processing, packaging, and transporting cover crop seeds are also not available and derived from maize seed production emissions and are expressed in kg CO_2e kg^{-1} of seed. The emissions of additional machinery operations for tillage, seeding, and mechanical termination of cover crops were included only in terms of additional diesel fuel usage. Average diesel fuel consumption of all additional operations was calculated and multiplied by the average GHG emissions of diesel fuel.

3. Results

The impact of all analyzed factors on the NCCMI of cover crops is summarized in Table . The results refer to a typical European crop rotation using a winter cover crop and maize as the following crop in spring. Individual impact factors are described in the following sections.

Table 3: Calculation of the net climate change mitigation impact of cover crops during an annual cropping cycle

	**EU-27 average per hectare (ha)**				**EU-27 total**			
	Unit	Mean	Low (-95% confidence level)	High (+95% confidence level)	Unit	Mean	Low (-95% confidence level)	High (+95% confidence level)
Area					1,000 ha	15,092.14	14,785.60	15,398.67
Average yield	Mg ha^{-1}	7.88	7.46	8.31				
Relative yield gain	%	8.8%	2.9%	14.8%				
Absolute yield gain	Mg ha^{-1}	0.70	0.21	1.23				
Carbon opportunity cost maize	kg CO_2e kg^{-1}	2.10	2.10	2.10				
**Carbon land benefit based on maize yield gain**	**Mg CO_2e ha^{-1}**	**1.46**	**0.45**	**2.59**	**1,000 Mg CO_2e**	**22,086.83**	**6,631.87**	**39,805.69**
**Carbon sequestration**	**Mg CO_2 ha^{-1}**	**1.43**	**0.86**	**2.01**	**1,000 Mg CO_2**	**21,632.51**	**12,701.51**	**30,915.61**
Nitrogen fixation	kg N ha^{-1}	52.49	32.06	72.92				
Reduced nitrogen leaching	kg N ha^{-1}	38.82	19.13	58.51				
Total nitrogen fertilizer savings	kg N ha^{-1}	91.32	51.19	131.44				
Nitrogen fertilizer emissions	kg CO_2e kg N^{-1}	11.23	9.02	13.43				
**Carbon benefit based on nitrogen fertilizer savings**	**Mg CO_2e ha^{-1}**	**1.03**	**0.46**	**1.77**	**1,000 Mg CO_2e**	**15,474.08**	**6,830.39**	**27,187.04**
**Reduced N_2O emissions due to less leaching**	**Mg CO_2e ha^{-1}**	**0.10**	**-0.15**	**0.35**	**1,000 Mg CO_2e**	**1,495.60**	**-2,218.43**	**5,362.37**
**Albedo change**	**Mg CO_2e ha^{-1}**	**0.20**	**-0.37**	**0.78**	**1,000 Mg CO_2e**	**3,087.10**	**-5,514.25**	**12,042.49**
**Total climate benefit**	**Mg CO_2e ha^{-1}**	~~4.23~~	**1.25**	**7.49**	**1,000 Mg CO_2e**	**63,776.11**	**18,431.09**	**115,313.18**
**N_2O emissions**	**Mg CO_2e ha^{-1}**	**0.04**	**-0.02**	**0.09**	**1,000 Mg CO_2e**	**555.77**	**-306.57**	**1,453.41**
**Foregone benefits due to cover crop seed land use**	**Mg CO_2e ha^{-1}**	**0.28**	**0.27**	**0.29**	**1,000 Mg CO_2e**	**4,180.56**	**3,970.76**	**4,395.55**
**Production emissions cover crop seed**	**Mg CO_2e ha^{-1}**	**0.39**	**0.08**	**0.70**	**1,000 Mg CO_2e**	**5,920.55**	**1,194.38**	**10,837.69**
**Processing, packaging, and transport of cover crop seed**	**Mg CO_2e ha^{-1}**	**0.08**	**0.08**	**0.08**	**1,000 Mg CO_2e**	**1,182.71**	**1,158.69**	**1,206.74**
Additional machinery operations	l Diesel ha^{-1}	45.29	32.44	58.13				
Diesel emissions	kg CO_2 l^{-1}	3.13	3.13	3.13				
**Additional machinery operations**	**Mg CO_2 ha^{-1}**	**0.14**	**0.10**	**0.18**	**1,000 Mg CO_2e**	**2,140.93**	**1,502.51**	**2,804.02**
**Total climate cost**	**Mg CO_2e ha^{-1}**	**0.93**	**0.51**	**1.34**	**1,000 Mg CO_2e**	**13,980.53**	**7,519.77**	**20,697.40**
**Net climate change mitigation impact of cover crops**	**Mg CO_2e ha^{-1}**	**3.30**	**0.74**	**6.14**	**1,000 Mg CO_2e**	**49,795.58**	**10,911.32**	**94,615.78**
Share of EU-27 agriculture emissions in 2020						13.0%	2.9%	24.7%

Note: Calculation, formulas and number of studies are provided as supplementary material (S1). Ha is hectares. EU-27 is the 27 Member States of the European Union (all Member States excluding the United Kingdom).

3.1. Carbon land benefit based on yield gain

The literature review on the impact of winter cover crop on maize yields revealed that yield effects are highly dependent on the cover crop species or the composition of the cover crop mixture. The yield effect of cover crops on the following crop is also highly dependent on the type of land management. The lower the tillage intensity, the higher the yield increase[18]. The yield increase effect is also much higher in organic farming than in conventional systems. Marcillo and Miguez[23] find a yield increase of 8.0% for a conventional intensive tillage system, while for an organic reduced tillage system, yields increased by 61.0% when cover cropping was implemented. In summary, the considered studies showed a weighted mean maize yield gain due to cover cropping of 8.8% (95% CI, 2.9, 14.8).

The following figure shows the included yield effect results, confidence intervals and the pooled effect. If more than one result is shown from one study in the figure this literature showed results from multiple experiments.

Fig. 1: Forest plot of included literature on yield effects

Multiplying the weighted mean yield gain with the average EU-27 maize yield and the carbon opportunity cost factor for maize (2,10 Kg CO_2e kg^{-1} maize[11]) results in a mean carbon land benefit increase of 1.46 Mg CO_2e ha^{-1}.

3.2. Carbon sequestration

Cover crops have a positive impact on soil organic carbon sequestration in arable soils. The literature research revealed two meta-analyses with 139 and 1195 studies included. In

addition, 26 studies that were not included in the above-mentioned meta-analyses were also included in our calculations. Up to 12.0% of carbon from cover crops can be converted to soil organic carbon[13]. Similar to the crop yield effects shown above, the potential of soil organic carbon stock increases depend on the type of cover crop. For example, a soil organic carbon increase of 0.26 Mg CO_2e ha^{-1} a^{-1} has been found for a winter vetch cover crop[13]. A much larger increase in soil carbon of 5.12 Mg CO_2e ha^{-1} a^{-1} was found ~~in~~ Abdalla et al.[24] for non-legume cover crops. The weighted mean of all studies investigated shows an average sequestration of 1.43 Mg CO_2e ha^{-1} a^{-1} (95% CI, 0.86, 2.01). Based on the included studies, grass cover crops lead to a 2.3-times higher soil organic carbon sequestration than legume cover crops. Cover crop mixtures of grasses, legumes, and various other species are in the middle of the field in sequestration performance[25].

The following figure shows the included carbon sequestration results, confidence intervals and the pooled effect. If more than one result is shown from one study in the figure this literature showed results from multiple experiments.

Fig. 2: Forest plot of included literature on carbon sequestration effects

3.3. Nitrogen fixation

Legume cover crops are able to fix atmospheric nitrogen and transfer it to the rhizosphere or utilize it for their own biomass. The decay of nitrogen-rich legume litter or rhizosphere products contribute to soil nitrogen fertilization and plant nutrition of the crop following the cover crop[13]. The amount of nitrogen fixed depends on the legume species and/or the companion crop species in the mixture. For example, red clover fixes 29.00 kg N ha^{-1} as a single species cover crop, but 52.00 kg N ha^{-1} in a mixture with ryegrass[13]. The same was observed

with winter vetch, which fixes 85.00 kg N ha^{-1} as single species and 113.00 kg N ha^{-1} in a mixture with winter rye[26]. A much wider range from 50.00 kg N ha^{-1} to 300.00 kg N ha^{-1} was found by Kaye and Quemada[21]. Wittwer et al.[18] showed that legumes as a single species cover crop can increase nitrogen uptake of maize by 32.00 kg N ha^{-1} and as part of cover crop mixtures by 28.00 kg N ha^{-1}. The summarized literature resulted in a weighted mean of 52.49 kg N ha^{-1} (95% CI, 32.06, 72.92) that can be contributed by legumes from nitrogen fixation.

3.4. Reduced nitrogen leaching

Cover crops prevent nutrient leaching over winter, especially of mineral nitrogen (N_{min}) that otherwise would be leached from the soil profile. However, this leaching reduction strongly depends on the timing of when cover crop are planted and type of cover crop. Early sown oil radish reduces leaching in the fall and winter months[27]. Rye as an over-wintering cover crop can better protect nitrogen from leaching in the spring[27]. Non-legume cover crops and mixtures can protect up to 100.00 kg N ha^{-1} from leaching, while pure legumes can protect between 60.00 to 70.00 kg N ha^{-1}[6,21]. Abdalla et al. [24]show that legume cover crops prevent 18.00 kg N ha^{-1} from leaching, non-legume cover crops 38.00 kg N ha^{-1}, and cover crop mixtures 19.00 kg N ha^{-1}. A study by McCracken et al.[28] resulted in an average leaching reduction of 35.80 kg N ha^{-1}. The amount of scavenged nitrogen depends heavily on water availability and soil type. Early sowing and sufficient precipitation enable cover crops to develop faster, establish more biomass, and absorb more nitrogen in the fall. The tillage system also affects the amount of scavenged nitorgen. The faster mineralization in conventional tillage systems in autumn resulted in higher N_{min} stocks and higher nitrogen uptake from cover crops than in no-till systems[29]. Literature research resulted in a weighted

mean value of 38.82 kg N ha^{-1} (95% CI, 19.13, 58.51) of scavenged nitrogen available to the crop following the cover crop.

3.5. Fertilizer emissions

Nitrogen fertilizer emissions are caused by different factors and vary between fertilizer types. About half of the CO_2e emissions of nitrogen fertilizer is N_2O emissions from soil after application and about one-third of all emissions occur during the production process of fertilizer itself. The remaining emissions are divided into N_2O emissions from NH_4^+ and NO_3^- losses and CO_2 from lime[30]. Total emissions per kg of nitrogen also vary between fertilizer types and the literature. For example, Hasler et al.[31] report 8.69 kg CO_2e kg^{-1} N for urea fertilizer to 11.64 kg CO_2e kg^{-1} N for $CaH_4N_4O_9$ fertilizer. A much higher emission factor of 15.00 to 31.00 kg CO_2e kg^{-1} N is used by Kahrl et al.[32]. The mean value of all studies considered was 11.23 kg CO_2e kg^{-1} N (95% CI, 9.02, 13.43). Emissions from organic fertilizers were not considered.

3.6. Reduced indirect N_2O emissions due to less leaching

Leached nitrogen is deposited into aquatic ecosystems and represents a globally significant source of N_2O through indirect emissions. These emissions can be calculated using the IPPC emission factor EF_5, which incorporates three components: EF_{5g} (groundwater and surface drainage), EF_{5r} (rivers) and EF_{5e} (estuaries). [33]

The IPPC emission factor EF_5 amounts to 0.0075 and is the sum of EF_{5g}, EF_{5r} and EF_{5e} with a value of 0.0025 each. Tian et al. [33] suggest a higher emission factor for EF_{5g} of 0.0060, the

other two factors are almost the same as those suggested by IPCC. Here we use the mean of two results: One using the IPPC factor which results in 0.08 Mg CO_2 -eq ha^{-1} and the other one using the factor of Tian et al.[33] which results in 0.12 Mg CO_2 -eq ha^{-1}. The mean of both values leads to an average climate benefit caused by reduced indirect N_2O emissions due to less leaching of 0.10 Mg CO_2 -eq ha^{-1} (95% CI, -0.15, 0.35).

3.7. Albedo change

When soil is covered with cover crops, less solar radiation reaches the soil surface compared to fallow land. Thus, the albedo value of plants is higher than that of fallow soil. Due to this reduced net radiation, there is a cooling effect that impacts the global climate. However, the albedo value of fallow soil strongly depends on soil type and condition. In particular, the color and moisture content of the soil play important roles. Dark and moist soils have a lower albedo than light dry soils, so there is a wide variation in the albedo value of fallow land. Since there is often a high soil moisture level during the growth period of cover crops (in fall and winter) the albedo effect of covering the soil with cover crops is stronger than for the yearly average[14]. Unfortunately, research on albedo effects of cover crops is in its infancy and only two adequate publications were found. The albedo effect of cover crops ranges from -4.00 to 10.00 W m^{-2} was calculated by Kaye and Quemada[21]. Converted to CO_2e, this corresponds to a climate benefit of 0.25 Mg CO_2e ha^{-1} a^{-1}. Carrer et al.[14] show that three months of cover cropping in Europe results in a climate benefit of 0.16 Mg CO_2e ha^{-1} a^{-1}. Similarly, this study found that longer time periods with cover cropping would increase the mitigation potential by 27.0%. Overall, a mean climate benefit of 0.20 Mg CO_2e ha^{-1} a^{-1} (95% CI, -0.37, 0.78) was identified in the considered literature for the albedo change of cover crops.

3.8. Nitrous oxide emissions

Cover cropping can increase N_2O emissions under certain weather conditions compared to fallow land. There are many factors that influence the level of N_2O emissions which leads to high variability from this GHG emission source. A prerequisite for cover crops is high soil moisture and water saturation, or at least water saturation of the upper soil layers. Another factor is the C/N ratio of the cover crop, which influences how fast cover crop residues decompose. Cover crops with low C/N ratios emit more N_2O than cover crops with high C/N ratios[34,35]. Another factor influencing N_2O emissions is the treatment of cover crop residues. Incorporating residues into the soil leads to higher N_2O emissions as compared to leaving cover crop residues decaying on the soil surface[34]. Air temperature also effects N_2O emissions. As temperatures increase, N_2O emissions from cover crops also increase. Overall, air temperature accounts for 22.0% of the variability in N_2O emissions[36]. In a meta-analysis, Basche et al.[37] found that N_2O emissions increased in 60.0% and decreased in 40.0% of the publications when cover cropping was practiced. The overall conclusion of the meta-analysis is that cover cropping increases N_2O emissions only to a very small extent and that the variability is very large. For example, compared to a chemical fallow system, Olofsson and Ernfors[38] ~~identify~~ the highest value with 1.80 kg N_2O-N ha^{-1} for oilseed radish, whereas Preza-Fontes et al.[39] ~~identify~~ the lowest emissions with -0.54 kg N_2O-N ha^{-1} for a sorghum-sudan-grass-mixture. The result of our calculation based on a systematic literature search also shows only a very small increase in N_2O emissions from cover cropping. The determined weighted mean value of emissions is 0.13 kg N_2O-N ha^{-1}, which is equivalent to 0.04 Mg CO_2e ha^{-1} (95% CI, -0.02, 0.09).

3.9. Foregone benefits due to cover crop seed land use

Land use for producing seeds for cover crops was calculated for the species phacelia, mustard, clover, and oat as representatives of the most important plant families used as cover crops. Their average was used for further calculations. The average land use requirement to produce one ha of cover crop seeds was calculated as 0.02 ha (95% CI, 0.00, 0.04).

We assume that the land used for the production of cover crop seeds would otherwise be used for wheat production. Wheat production on land in the EU offers a carbon benefit that consists of: (1) the opportunity that the wheat output enables storing carbon elsewhere (yield × carbon opportunity cost); and (2) savings in global production emissions due to lower production emissions in the EU compared to the global average (here, the difference between Swedish and global wheat production emissions is based on Searchinger et al.[11]). Other factors, like changes in soil organic carbon or bioenergy benefits are not considered here. The climate benefit of wheat consisting of factors (1) and (2) is forgone due to land requirements for cover crops' seed requirements and can be quantified accordingly to a mean value of 0.28 Mg CO_2e ha^{-1} (95% CI, 0.27, 0.29).

3.10. Production emissions for cover crop seed production

Since no specific literature sources for seed production emissions could be found for typical cover crop species, emissions from corn, spring wheat, and canola seed production are used in this study. Hybrid corn seed production in China leads to emissions of 1,459.00 kg CO_2e Mg^{-1}, whereas in the U.S. it causes 2,250.50 kg CO_2e Mg^{-1}[17,40] of emissions. Seed production of rapeseed in Poland causes slightly lower emissions of 1,014.00 kg CO_2e Mg^{-1}[41]. In contrast,

for spring wheat, it is only 580.00 kg CO_2e Mg^{-1} in Southwest Finland and 680.00 kg CO_2e Mg^{-1} in Northern Savonia (Finland)[42]. These emissions mostly depend on the amount of fertilizer used since fertilization is responsible for the majority of production emissions, especially the production and application of nitrogen fertilizers[17]. The weighted average value used for our calculation is 1,066.26 kg CO_2e Mg^{-1} (95% CI, 219.56, 1912.95). For the emissions of processing, packaging, and transporting cover crop seeds, only one literature source for corn seed could be found. The emissions here are 213.00 kg CO_2e Mg^{-1}[43].

3.11. Additional machinery operations for cover crop establishment

We assume that cover crop establishment requires either tillage with a plow or cultivator followed by seeding. In addition, it is assumed that the only relevant emission from these machinery operations is from burning fossil diesel fuel. Based on data from KTBL[44], plowing plus seeding is estimated at 55.00 l diesel fuel ha^{-1}, while cultivator tillage and seeding is estimated at 45.00 l diesel fuel ha^{-1}. The variability of the machinery operation intensity and corresponding fuel consumption was analyzed by Wittwer et al.[18]. In this study, diesel fuel consumption ranges from 22.00 l ha^{-1} for no-till systems to 61.00 l ha^{-1} for conventional intensive tillage systems. In organic farming, diesel fuel consumption ranges from 32.00 l ha^{-1} for reduced tillage to 55.00 l ha^{-1} for intensive tillage. The average diesel fuel consumption used in this study was calculated as 45.29 l ha^{-1} (95% CI, 32.43, 58.13).

The emission factor per liter of diesel fuel ranges from 3.00 kg CO_2e l^{-1}[43] to 3.31 kg CO_2e l^{-1}[45]. The average emissions were calculated as 3.13 kg CO_2e l^{-1} (95% CI, 2.92, 3.34) diesel fuel.

The additional machinery operations thus cause emissions of 0.14 Mg CO_2e ha^{-1} (95% CI, 0.10, 0.18).

3.12. A factor not considered: erosion reduction

According to Lugato et al.[46], soil erosion can lead to a loss of soil carbon, lowering the carbon sink capacity of soils. Additionally, soil erosion can disturb the soil structure and reduce soil fertility, which disconnects soil element cycles and further contributes to GHG emissions. However, the extent to which soil erosion leads to higher carbon fluxes out of the system depends on the specific context and interplay of many factors[47].

Cover crops protect soil erosion during the fall and winter and also after the establishment of the following crop, such as maize, due to remaining cover crop residues[48]. According to Laloy and Bielders[48] and Gentsch et al.[6], Cover crops increase the infiltration rate and improve soil structure, allowing the soil to absorb more water without eroding. According to Panagos et al.[15], cover crops reduce soil erosion by at least 20.0% in Europe and the United Kingdom. Machiwal et al.[49] found a reduction in soil loss of 33.0% to 77.0%, depending on the cover crop type for India. The reduction in soil erosion is highly dependent on the slope and management of the land. In addition, the soil type also plays a major role.

Given the uncertainty between soil erosion and fluxes from the soil system as well as the varying factors leading to soil erosion, we have not included climate benefits of soil erosion prevention from cover crops in our calculations.

3.13. Net climate change mitigation impact (NCCMI) of cover crops

We calculate the summary of all climate benefits from cover crops as 4.23 Mg CO_2e ha^{-1} a^{-1} (95%CI, 1.25, 7.49) and all climate costs from cover crops as 0.93 Mg CO_2-eq ha^{-1} a^{-1} (95% CI 0.51, 1.34). The NCCMI of cover crops was calculated as 3.30 Mg CO_2e ha^{-1} a^{-1} (Table). The NCCMI was then extrapolated to the maize harvest area for all EU-27 countries. Here, we assume a scenario in which cover crops are grown before maize on all of the EU-27's harvested maize areas, which is 15,092 x 10^6 ha, on average[22]. Based on this calculation, planting cover crops before maize in the EU-27 results in a climate change mitigation potential of 49.80 Mio. Mg CO_2e a^{-1}. This is equivalent to 13.0% of the EU-27's agricultural GHG emissions[50].

4. Discussion

4.1. Review of results

The results of this study indicate that the climate benefits of cover crops significantly exceed their climate costs. This resulted in a positive NCCMI of 3.30 Mg CO_2e ha^{-1} a^{-1} that can be achieved if cover crops were incorporated in crop rotations of maize production. The major contributor to climate benefits is through carbon land benefits based on yield gain for maize following a cover crop, which contributed to 33.8% of all climate benefits. This is the first study to include this effect of cover crops on climate change mitigation. Several meta studies[9,10] ~~show~~ that cover crops increase soil organic carbon stocks, with an average organic carbon sequestration rate of 1.43 Mg ha^{-1} a^{-1}. The most important mechanism behind this is the

stimulation of microbial activity from cover crops' organic matter input. Litter from shoots and roots, rhizodeposits, and the transport of photoassimilate to the rhizospher during the growth of cover crops results in a higher and more active microbial biomass[51]. These processes resulted in microbial derived organic substances that are key to build up mineral associated organic matter fractions with prolonged turnover times[52]. The activation of microbial cycling that derived from higher organic matter input rates is therefore a strong indirect factor of the climate benefits from cover crops. Carbon sequestration was the second largest contributor (34.5%) to climate benefits. The results indicate a maize yield increase of 8.8% following a cover crop. Since maize yield gains are higher following legume cover crops and in organic systems, it can be assumed that increased nutrient availability is one of the key factors driving crop yield gains from cover crops. Cover crops might help in closing the yield gap between organic and conventional systems, as well as in improving the resilience of arable production under climate change. However, yield benefits depend on the quality of cover crop residues and reach its maximum at litter C/N ratios <25[53]. Closer nutrient cycling and reduction of leaching losses resulted in another 24.3% of cover crops' climate benefits, including nitrogen fixation of legume cover crops. The highest maize yield gains were found for legume-based cover crops while graminoid cover crops showed only minor effects. For example, Marcillo and Miguez[23] found that a grass cover crop has no influence on maize yield. In the same study, a legume-only mixture shows up to a 21.0% yield gain in the maize crop following the cover crop. Wittwer et al.[18] ~~confirm~~ that a legume-free cover crop mixture leads to only a 3.0% increase in maize yield. Mixtures of legumes and grasses as cover crops enable yield gains from 1.3%[54] to 21.0%[55], respectively. However, cover crops can also have negative effects on the subsequent maize yield. Hunter et al.[56] ~~show~~ that a high carbon to nitrogen (C/N) ratio in spring cover crops resulted in a lower yield of silage maize.

Nutrient use efficiency has been demonstrated to increase through cover crops that are in crop rotations for several crops, including maize[57–59]. The inclusion of legumes increase cover crops' litter quality and crop yield benefits; however, pure legume stands are less effective to prevent nitrogen leaching over the winter[6]. Including cover crops in crop rotations, therefore, requires a high degree of management by selection of suitable cover crop species or mixtures to maximize their green manure and environmental benefits.

Cover crops are able to change the amount of incoming shortwave radiation that is reflected back to the atmosphere and thereby mitigate warming[21]. Albedo change through cover crops' land cover has the smallest contribution (4.5%) to the climate benefits from cover crops. The systematic literature search resulted in only two publications that analyze albedo change through cover crops. Both found a slight climate change mitigating effect through radiative forcing changes induced by an increase in surface albedo.

Climate costs of cover crops are nearly six times lower than climate benefits and are mainly dominated by cover crops' seed production emissions and foregone benefits due to cover crops' seed land use (accounting for 41.9% and 30.1%, respectively, of the total climate costs of cover crops). Interestingly, additional machinery operations and N_2O emissions from cover crops' decomposition contributed minorly to climate costs (15.0% and 4.3%, respectively). Emissions of N_2O have a 280 times higher global warming potential compared to CO_2. Therefore, the latest studies were alarmed about the emission potential that can appear from decomposition of cover crop residues[37]. However, N_2O emissions are of episodic nature and depend on extended precipitation events. Despite this, N_2O emissions from cover crops' decomposition can be further reduced if cover crop residues were not incorporated into the soil[37]. With this respect, no-till operations would further reduce the N_2O emissions factor.

4.2. Sensitivity analysis based NCCMI scenarios

The impact factors “carbon land benefit based on yield gain” and “carbon sequestration” have a high impact on NCCMI but also show a high uncertainty. To address this uncertainty, we use four scenarios for NCCMI to analyze the sensitivity of the results.

These four scenarios are:

a) Base scenario as shown in section 3.
b) Base scenario less carbon land benefit based on yield gain
c) Base scenario less carbon sequestration
d) Base scenario less carbon land benefit based on yield gain and less carbon sequestration

Following figure shows the absolute NCCMI results of the scenarios per hectare (left) and the relative NCCMI result as percentage of total EU agricultural emissions in 2020 (right):

Figure 3: Sensitivity analysis of NCCMI scenarios as absolute results per ha (left) and relative results compared to EU ag. emissions (right)

All scenarios show a positive mean value. The 95% confidence levels of scenario A and B indicate that the values are statistically significant different from zero at the 0.05 level. The confidence levels of scenario C and D indicate that the values are not different from zero at the 0.05 level.

4.3. Comparing overall results to existing literature

In contrast to Kaye and Quemada[21], this study is first to include land use effects based on the yield gain caused by cover crops. Furthermore, our study uses a systematic literature review and quantifies the climate change mitigation impact for all of EU-27, which demonstrates the magnitude of climate change mitigation opportunities of cover crops. So far no other literature has extrapolated the climate change mitigation impact of cover crops to a country or region. Overall, Kaye and Quemada[21] found a climate change mitigation effect of 1.16 - 1.35 Mg CO_2e ha^{-1} a^{-1} from cover crops, which is lower than that calculated in this study. This discrepancy is a result of different analyzed literature and the inclusion of land use effects in our research. In addition, Abdalla et al.[24] ~~show~~ a slightly lower climate change mitigation impact from cover crops compared to our results of 2.06 Mg CO_2e ha^{-1} a^{-1}(95% CI -0.04, 4.16). The study, however, does not consider land use change effects (1.51 Mg CO_2e ha^{-1} a^{-1} in our study), albedo change, emissions for cover crop seed production, and additional machinery operations. If the study would have considered these factors, their results would be more comparable to those in our study.

4.4. Limitations

A limitation of this study is that we did not include erosion reduction of cover crops as a climate benefit due to the high data variability and uncertainty of its climate impact. Furthermore, the use of carbon opportunity cost as suggested by Searchinger at al.[11] is criticized by some authors for separating supply and demand side efficiencies and including supply and demand side interactions[60,61]. Despite this criticism, carbon opportunity cost is

a widely used approach to quantify changes in efficiency of land use for mitigating climate change. Given that land use change is still the highest source of emissions in the global agri-food sector[2], carbon opportunity cost based on Searchinger at al.[11] is an adequate land use change quantification approach for changes in maize yield.

For the climate impact of land use for cover crop seed production, we use the forgone climate benefit of wheat based on Searchinger et al.[11]. Here, the same arguments discussed above for using carbon opportunity costs are relevant. We assume wheat as the displaced crop when cover crop seeds are produced since wheat is the crop with the largest acreage in the European Union[22]. For additional machinery operations to establish cover crops, we only consider diesel fuel emissions. There are also emissions for producing, maintaining, and repairing agricultural machinery, but in the majority of cases, existing machinery is used for cover crop establishment, i.e., no additional machinery is produced to establish cover crops.

In this study, we only evaluated maize as the crop to follow cover crops. Maize is the largest spring crop by acreage in the European Union[22] and the most investigated crop in the scientific literature with a good data base. Despite this, other spring crops like oats, spring barley, sunflower, or sugar beet are investigated for the benefits of winter cover crops in the European Union. More data is needed to complete the view on climate benefits of cover crops based on different main crops.

4.5. Policy recommendations

This is the first study to include all relevant climate change impacting effects of cover crops in a comprehensive calculation and to extrapolate the NCCMI of cover crops to the whole maize cropping area of the EU-27. We show that cover crops are powerful tools to mitigate climate

change impacts from European agriculture, which make our findings very relevant for political decisions in the EU. If all maize cropping acreage in the EU-27 were to include cover crops, GHG emissions from EU-27 agriculture could be reduced by 13.0%. Based on this result, we recommend that the Common Agricultural Policy (CAP) of the European Union should continue to accelerate the integration of cover crops into farming. The current CAP (2023–2027) does not include regulations that make cover crops before spring crops mandatory or provide incentives so the area devoted to cover crops in the EU is maximized. There are conditionality requirements, such as obligatory soil cover in the winter for 80% of a farm's arable land. Therefore, if a farm has 80% winter crops and 20% spring crops in its rotation, there is no requirement to grow cover crops under the current CAP for this farm[62].

Our results recommend a stronger commitment from the EU's agricultural policy to make cover crops before spring crops a common practice on all farms. This will not only help achieve the EU's climate protection goals, but also global targets in the Paris Agreement.

Statistics

The weighted mean was calculated from study values using the number of observations as the weighting factor. Mean values are followed by the 95% confidence intervals (CI) and are shown in brackets (lower CI limit, upper CI limit). All data collected from the literature as well as the calculations shown in Table are provided as supplementary material (S1).

Acknowledgements

We would like to thank the German Federal Ministry of Education and Research for supporting this research through funding the project CATCHY (catch-cropping is an agrarian tool for continuing soil health and yield-increase).

Author contributions

JS: Writing, investigation, data curation, formal analysis

NG: Writing, investigation, methodology, funding acquisition

PB: Writing, conceptualization, supervision, funding acquisition

Data availability statement

The reference list from the literature research is provided in the supplementary material (S1). All data extracted are provided as an Excel file in the supplementary material (S2).

Additional Information

The authors declare no conflict of interest.

References

1. Clark MA, Domingo NGG, Colgan K, Thakrar SK, Tilman D et al. (2020) Global food system emissions could preclude achieving the 1.5° and 2°C climate change targets. Science (New York, N.Y.) 370 (6517): 705–708.
2. Crippa M, Solazzo E, Guizzardi D, Monforti-Ferrario F, Tubiello FN et al. (2021) Food systems are responsible for a third of global anthropogenic GHG emissions. Nat Food 2 (3): 198–209.

3. Shukla PR, Skea J, Reisinger A, editors (2022) Climate change 2022. Mitigation of climate change. Geneva: IPCC. 1977 p.
4. Lal R (2020) Managing soils for negative feedback to climate change and positive impact on food and nutritional security. Soil Science and Plant Nutrition 66 (1): 1–9.
5. Thorup-Kristensen K, Magid J, Jensen LS (2003) Catch crops and green manures as biological tools in nitrogen management in temperate zones. Advances in Agronomy Volume 79: Elsevier. pp. 227–302.
6. Gentsch N, Heuermann D, Boy J, Schierding S, Wirén N von et al. (2022) Soil nitrogen and water management by winter-killed catch crops. SOIL 8 (1): 269–281.
7. Kim N, Zabaloy MC, Guan K, Villamil MB (2020) Do cover crops benefit soil microbiome? A meta-analysis of current research. Soil Biology and Biochemistry 142: 107701.
8. Hudek C, Putinica C, Otten W, Baets S de (2022) Functional root trait-based classification of cover crops to improve soil physical properties. European J Soil Science 73 (1).
9. Christopher Poeplau, Axel Don (2015) Carbon sequestration in agricultural soils via cultivation of cover crops - A meta-analysis. Agriculture, Ecosystems and Environment 200: 33–41.
10. Jinshi Jian, Xuan Du, Mark S Reiter, Ryan D Stewart (2020) A meta-analysis of global cropland soil carbon changes due to cover cropping.
11. Timothy D. Searchinger, Stefan Wirsenius, Tim Beringer, Patrice Dumas (2018) Assessing the efficiency of changes in land use for mitigating climate change. Nature 564 (7735): 249–253.
12. Emma Kovak, Dan Blaustein-Rejto, Matin Qaim (2022) Genetically modified crops support climate change mitigation. Trends in Plant Science. Available: https://linkinghub.elsevier.com/retrieve/pii/S1360138522000048.
13. Chiara De Notaris, Jørgen Eivind Olesen, Peter Sørensen, Jim Rasmussen (2020) Input and mineralization of carbon and nitrogen in soil from legume-based cover crops. Nutrient Cycling in Agroecosystems 116 (1).
14. Carrer D, Pique G, Ferlicoq M, Ceamanos X, Ceschia E (2018) What is the potential of cropland albedo management in the fight against global warming? A case study based on the use of cover crops. Environ. Res. Lett. 13 (4): 44030.
15. Panos Panagos, Cristiano Ballabio, Mihaly Himics, Simone Scarpa, Francis Matthews et al. (2021) Projections of soil loss by water erosion in Europe by 2050. Environmental Science & Policy 124: 380–392.
16. van Groenigen JW, Huygens D, Boeckx P, Kuyper TW, Lubbers IM et al. (2015) The soil N cycle: new insights and key challenges. SOIL 1 (1): 235–256.
17. Dan Liu, Wushuai Zhang, Xiaozhong Wang, Yanjun Guo (2021) Greenhouse gas emissions and mitigation potential of hybrid maize seed production in northwestern China.
18. Raphaël A. Wittwer, Brigitte Dorn, Werner Jossi, Marcel G. A. van der Heijden (2017) Cover crops support ecological intensification of arable cropping systems. Scientific Reports 7 (1): 41911. Available: http://www.nature.com/articles/srep41911.
19. Li J, Xie RZ, Wang KR, Ming B, Guo YQ et al. (2015) Variations in Maize Dry Matter, Harvest Index, and Grain Yield with Plant Density. Agronomy Journal 107 (3): 829–834.
20. IPPC (2006) N2O EMISSIONS FROM MANAGED SOILS, AND CO 2 EMISSIONS FROM LIME AND UREA APPLICATION. Available: https://www.ipcc-nggip.iges.or.jp/public/2006gl/pdf/4_Volume4/V4_11_Ch11_N2O&CO2.pdf. Accessed 21 November 2023.

21. Jason P. Kaye, Miguel Quemada (2017) Using cover crops to mitigate and adapt to climate change. A review. Agronomy for Sustainable Development 37 (1): 4. Available: http://link.springer.com/10.1007/s13593-016-0410-x.
22. Eurostat (2023) The official portal for European data. Available: https://data.europa.eu/data/datasets?locale=en. Accessed 11 November 2022.
23. G S Marcillo, F E Miguez (2017) Corn yield response to winter cover crops: An updated meta-analysis. Journal of Soil and Water Conservation 72 (3): 226–239. Available: www.swcs.org.
24. Mohamed Abdalla, Astley Hastings, Kun Cheng, Qian Yue, Dave Chadwick, Mikk Espenberg, Jaak Truu, Robert M Rees, Pete Smith (2019) A critical review of the impacts of cover crops on nitrogen leaching, net greenhouse gas balance and crop productivity. Global Change Biology 25. 2530-2543 p.
25. Sabrina J. Ruis, Humberto Blanco-Canqui (2017) Sci-Hub | Cover Crops Could Offset Crop Residue Removal Effects on Soil Carbon and Other Properties: A Review. Agronomy Journal, 109(5), 1785 | 10.2134/agronj2016.12.0735. Available: https://sci-hub.se/10.2134/agronj2016.12.0735.
26. Chiara De Notaris, Jørgen Eivind Olesen, Peter Sørensen, Jim Rasmussen (2020) Input and mineralization of carbon and nitrogen in soil from legume-based cover crops. Nutrient Cycling in Agroecosystems 116 (1).
27. Gaimaro J, Timlin D, Tully K (2022) Comparison of cover crop monocultures and mixtures for suppressing nitrogen leaching losses. Agricultural Water Management 261: 107348.
28. McCracken DV, Smith MS, Grove JH, MacKown CT, Blevins RL (1994) Nitrate Leaching as Influenced by Cover Cropping and Nitrogen Source. Soil Science Society of America Journal 58 (5): 1476–1483.
29. Ritter WF, Scarborough RW, Chirnside A (1998) Winter cover crops as a best management practice for reducing nitrogen leaching. Journal of Contaminant Hydrology 34 (1-2): 1–15.
30. Frank Brentrup, Antoine Hoxha, Bjarne Christensen (2016) Carbon footprint analysis of mineral fertilizer production in Europe and other world regions.
31. K. Hasler, S. Bröring, S. W.F. Omta, H. W. Olfs (2015) Life cycle assessment (LCA) of different fertilizer product types. European Journal of Agronomy 69: 41–51.
32. Fredrich Kahrl, Yunju Li, Yufang Su, Timm Tennigkeit, Andreas Wilkes et al. (2010) Greenhouse gas emissions from nitrogen fertilizer use in China. Environmental Science & Policy 13 (8): 688–694.
33. Tian L, Cai Y, Akiyama H (2019) A review of indirect N2O emission factors from agricultural nitrogen leaching and runoff to update of the default IPCC values. Environmental Pollution 245: 300–306.
34. Guenet B, Gabrielle B, Chenu C, Arrouays D, Balesdent J et al. (2021) Can N2 O emissions offset the benefits from soil organic carbon storage. Global Change Biology 27 (2): 237–256.
35. Hélène Tribouillois, Julie Constantin, Eric Justes (2018) Cover crops mitigate direct greenhouse gases balance but reduce drainage under climate change scenarios in temperate climate with dry summers. Global Change Biology 24 (6): 2513–2529.
36. Ihsan Muhammad, Upendra M. Sainju, Fazhu Zhao, Ahmad Khan, Rajan Ghimire et al. (2019) Regulation of soil CO2 and N2O emissions by cover crops: A meta-analysis. Soil and Tillage Research 192 (April): 103–112.

37. Basche AD, Miguez FE, Kaspar TC, Castellano MJ (2014) Do cover crops increase or decrease nitrous oxide emissions? A meta-analysis. Journal of Soil and Water Conservation 69 (6): 471–482.
38. Olofsson F, Ernfors M (2022) Frost killed cover crops induced high emissions of nitrous oxide. The Science of the total environment 837: 155634.
39. Preza-Fontes G, Tomlinson PJ, Roozeboom KL, Warren J, Ruiz Diaz DA (2020) Nitrogen fertilization offsets the N 2 O mitigating effects of cover-crops and double-crop soybean in a wheat–sorghum system. Agronomy Journal 112 (2): 772–785.
40. Seungdo Kim, Bruce E. Dale, Pam Keck (2014) Energy Requirements and Greenhouse Gas Emissions of Maize Production in the USA. Bioenergy Research 7: Springer New York LLC. 753-764 p.
41. M Borzęcka-Walker, A Faber, Z Jarosz, A Syp, R Pudełko (2005) WFL Publisher Science and Technology Greenhouse gas emissions from rape seed cultivation for FAME production in Poland. Journal of Food, Agriculture & Environment 11. Available: www.world-food.net.
42. Joensuu K, Rimhanen K, Heusala H, Saarinen M, Usva K et al. (2021) Challenges in using soil carbon modelling in LCA of agricultural products—the devil is in the detail. Int J Life Cycle Assess 26 (9): 1764–1778.
43. Effenberger Mathias, Gödeke Katja, Grebe Sven, Haenel Hans-Dieter, Hansen Anja, Häußermann Uwe, Kätsch Stepahnie, Lasar Ansgar, Nyfeler-Brunner Aurelia, Osterburg Bernhard, Paffrath Petra, Poddey Eike, Schmid Harald, Schraml Martine, Wulf Sebastian, Zerhusen Bianca (2016) Berechnungsstandard für einzelbetriebliche Klimabilanzen (BEK) in der Landwirtschaft. Available: https://www.ktbl.de/fileadmin/user_upload/Allgemeines/Download/BEK/Handbuch.pdf. Accessed 19 July 2022.
44. KTBL (2023) KTBL-Dieselbedarfsrechner. Available: https://daten.ktbl.de/dieselbedarf/home.html. Accessed 20 July 2022.
45. Umweltbundesamt (2019) Berechnung von Treibhausgas (THG)-Emissionen verschiedner Energieträger. Available: https://secure.umweltbundesamt.at/co2mon/co2mon.html. Accessed 23 July 2022.
46. Lugato E, Smith P, Borrelli P, Panagos P, Ballabio C et al. (2018) Soil erosion is unlikely to drive a future carbon sink in Europe. Science advances 4 (11): eaau3523.
47. Blanco-Canqui H, Shaver TM, Lindquist JL, Shapiro CA, Elmore RW et al. (2015) Cover Crops and Ecosystem Services: Insights from Studies in Temperate Soils. Agronomy Journal 107 (6): 2449–2474.
48. Eric Laloy, C. L. Bielders (2010) Effect of Intercropping Period Management on Runoff and Erosion in a Maize Cropping System. Journal of Environmental Quality 39 (3): 1001–1008.
49. Deepesh Machiwal, Sushil Kumar, Adlul Islam, Sanjay Kumar, Sita Ram Jat et al. (2021) Evaluating effect of cover crops on runoff, soil loss and soil nutrients in an Indian arid region. Available: https://www.tandfonline.com/action/journalInformation?journalCode=lcss20.
50. EEA (2021) EEA greenhouse gases — data viewer. Data viewer on greenhouse gas emissions and removals, sent by countries to UNFCCC and the EU Greenhouse Gas Monitoring Mechanism (EU Member States). Available: https://www.eea.europa.eu/data-and-maps/data/data-viewers/greenhouse-gases-viewer. Accessed 7 April 2023.

51. Gentsch N, Boy J, Batalla JDK, Heuermann D, Wirén N von et al. (2020) Catch crop diversity increases rhizosphere carbon input and soil microbial biomass. Biol Fertil Soils 56 (7): 943–957.
52. Kleber M, Eusterhues K, Keiluweit M, Mikutta C, Mikutta R et al. (2015) Mineral–Organic Associations: Formation, Properties, and Relevance in Soil Environments: Elsevier. pp. 1–140.
53. Finney DM, White CM, Kaye JP (2016) Biomass Production and Carbon/Nitrogen Ratio Influence Ecosystem Services from Cover Crop Mixtures. Agronomy Journal 108 (1): 39–52.
54. CUTTI L, LAMEGO FP, AGUIAR ACM de, KASPARY TE, RIGON CAG (2016) WINTER COVER CROPS ON WEED INFESTATION AND MAIZE YIELD. Rev. Caatinga 29 (4): 885–891.
55. Decker AM, Clark AJ, Meisinger JJ, Mulford FR, McIntosh MS (1994) Legume Cover Crop Contributions to No-Tillage Corn Production. Agronomy Journal 86 (1): 126–135.
56. Mitchell C Hunter, Meagan E Schipanski, Macdonald H Burgess, James C Lachance, Brosi A Bradley et al. (2019) Cover Crop Mixture Effects on Maize, Soybean, and Wheat Yield in Rotation: 1–5.
57. Chinta YD, Uchida Y, Araki H (2020) Availability of nitrogen supply from cover crops during residual decomposition by soil microorganisms and its utilization by lettuce (Lactuca sativa L.). Scientia Horticulturae 270: 109415.
58. Mahama GY, Prasad PVV, Roozeboom KL, Nippert JB, Rice CW (2016) Response of Maize to Cover Crops, Fertilizer Nitrogen Rates, and Economic Return. Agronomy Journal 108 (1): 17–31.
59. Sabet M, Majnoun Hosseini N, Rahimian Mashhadi H, Asadi S (2022) Potato Yield Improvement Through Integrated Cover Cropping and Nitrogen Management Occurred in Semiarid Climatic Condition. Potato Res. 65 (4): 959–977.
60. Eyhorn F, Muller A, Reganold JP, Frison E, Herren HR et al. (2019) Sustainability in global agriculture driven by organic farming. Nat Sustain 2 (4): 253–255.
61. van der Werf HMG, Knudsen MT, Cederberg C (2020) Towards better representation of organic agriculture in life cycle assessment. Nat Sustain 3 (6): 419–425.
62. European Commission (2022) COMMON AGRICULTURAL POLICY FOR 2023-2027. 28 CAP STRATEGIC PLANS AT A GLANCE. Available: https://agriculture.ec.europa.eu/system/files/2022-12/csp-at-a-glance-eu-countries_en.pdf. Accessed 3 May 2023.

Fig. 1

Click here to access/download;Figure;S1 Fig1.png

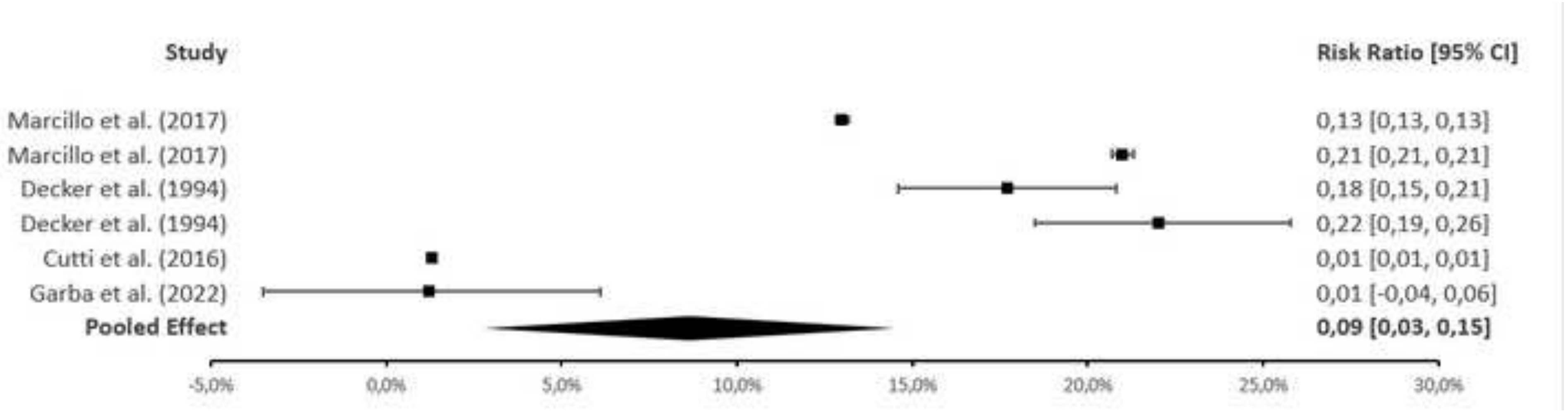

Fig. 2

Click here to access/download;Figure;S2 Fig2.png

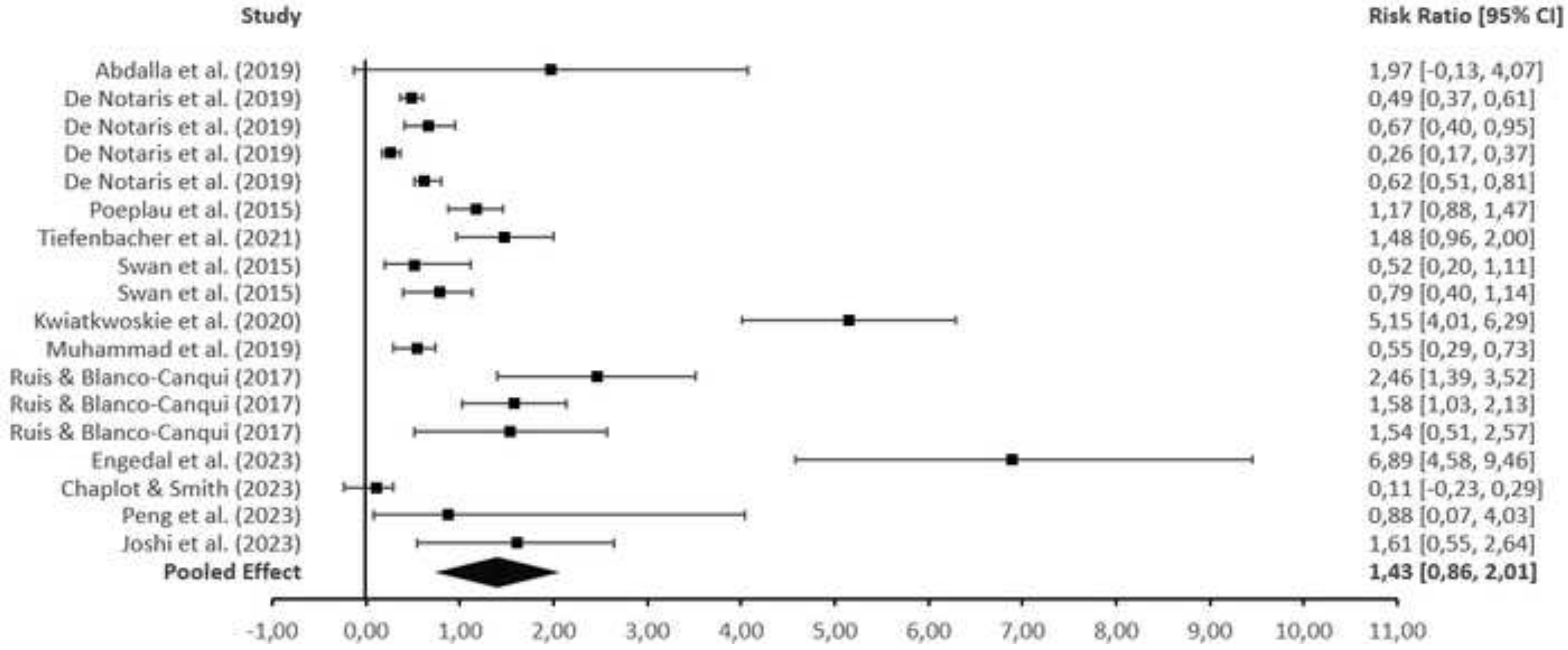

Fig. 3

Click here to access/download;Figure;S3 Fig3.png

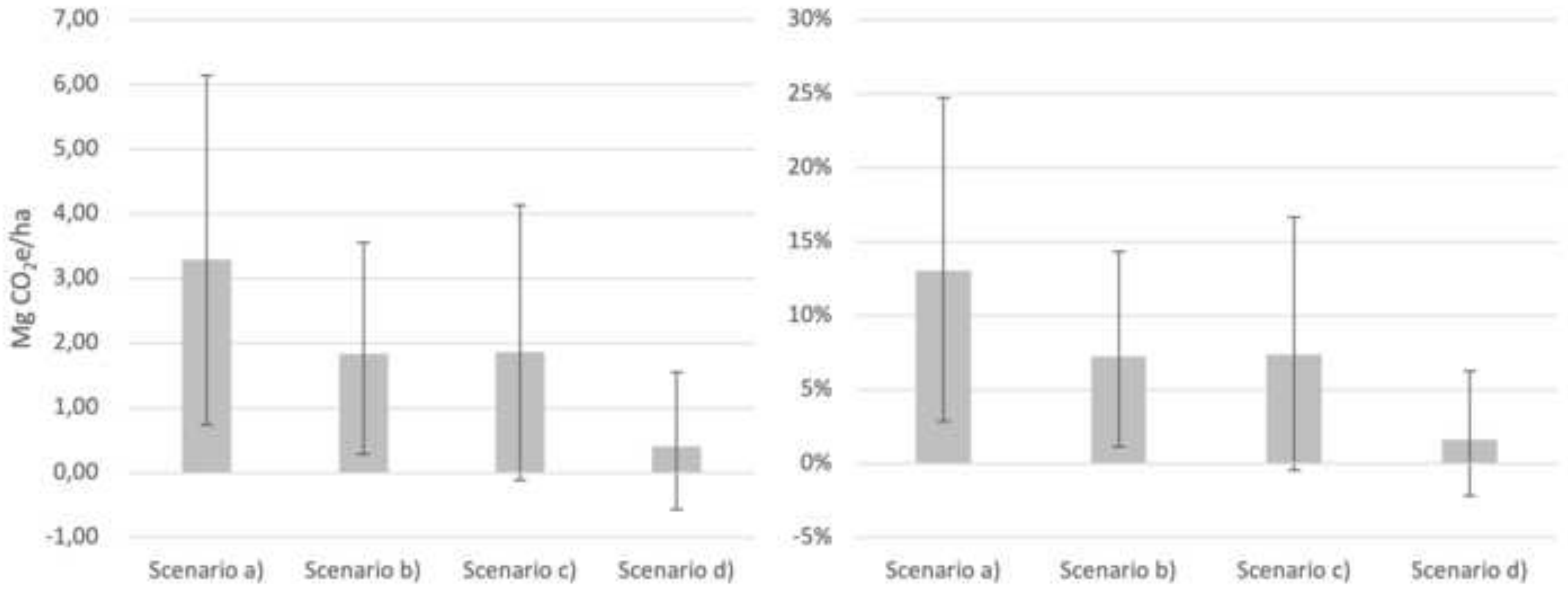

Calculation File

Click here to access/download
Supporting Information
S4 Calculations.xlsx

Included Literature

Click here to access/download
Supporting Information
S5 Included Literature.xlsx
