# Supplementary material for: Cover crops support the climate change mitigation potential of agroecosystems
Source: PLoS One. 2024 May 8;19(5):e0302139. doi: 10.1371/journal.pone.0302139 (PMC11078372; doi:10.1371/journal.pone.0302139)
Supplement: Attachment — Submitted filename: Response to Reviewers.pdf [file pone.0302139.s006.pdf]

WEIHENSTEPHAN · TRIESDORF
University of Applied Sciences

Hochschule Weihenstephan-Triesdorf | 91746 Weidenbach

Abhay Omprakash Shirale, PhD, Academic Editor

Dr. Vasudev Meena, Dr. S.D. Jadhao, Reviewers

PLOS ONE

Response to Reviewers for Manuscript "Cover crops support the climate change mitigation potential of agroecosystems"

Triesdorf, 01.03.2024

Faculty of Agriculture, Food and Nutrition

Prof. Dr. Peter Breunig
T +49 9826 654-217
F +49 9826 654-4217
peter.breunig@hswt.de

Hochschule
Weihenstephan-Triesdorf
University of Applied Sciences
Markgrafenstr. 16
91746 Weidenbach
Germany
www.hswt.de

Dear Academic Editor and Reviewers,

Thank you for your supportive and insightful review of our manuscript "Cover crops support the climate change mitigation potential of agroecosystems". In the following we would like to respond to each point raised by the academic editor and reviewers:

Journal Requirements

We went through all five journal requirements and can confirm that the reviewed manuscript fulfills all the raised points.

Comments of the Reviewers

- *Clear take home message in the abstract is required.*
  The abstract has been adjusted accordingly.
- *Abstract does not provide sufficient information about the literature review study.*
  The abstract has been adjusted accordingly.
- *The Introduction is well written but does not present the reader with sufficient information on the problem being addressed.*
  We added more clarity about the research problem in the introduction.
- *What are the objectives of this literature review study?*
  We added more clarity about the research problem in the introduction.
- *The methods are largely well written. Although details of the methods adopted for this study provided in separate file, same is required to include in the methodology section.*
  More details on the calculation method that is used for calculating maize yield effects were added to the manuscript.

- *The Statistical Analysis is pretty much absent.*
  We added a detailed statistical analysis including forest plots for yield gain and carbon sequestration. Due to limited data, a statistical analysis is not feasible for the other impact factors. In addition, we added Sankey plots for the described scenarios (Fig. 3).
- *The paper has no Conclusion and just kind of ends as per specific Journal style, instead author provided policy recommendations.*
  We changed the chapter 4.5. to “Conclusion and policy recommendations”.
- *Line 148.*
  Text has been changed accordingly.
- *Line 239 Production emissions for seed production.*
  Text has been changed accordingly.
- *Line 630 Follow reference formatting as per Journal guidelines.*
  Text has been changed accordingly.
- *Line 13 whether experiment conducted or its review study?*
  Text has been adjusted accordingly.
- *Line 91 References to support the information provided in table?*
  Reference has been added.
- *Line 178 Reduced N2O-N= EF5*Reduced N Leaching * 273 1000... Supporting references?*
  Reference has been added.
- *Line 233 & 234 Not understand what subtitle author want to represent.*
  Text has been changed accordingly.
- *Line 253 How relative yield gain and absolute yield gain estimated? Explain in methods section.*
  Text has been changed accordingly.
- *Line 280 Not understand?*
  Text has been changed accordingly.
- *Line 428 & 429*
  Text has been changed accordingly.
- *Line 477*
  Text has been changed accordingly.

All other comments that were provided directly in the manuscript (pdf) by the reviewers were adjusted as suggested.

Again, we would like to thank you for your review comments and suggestions to our manuscript. We believe that the revised manuscript provides more clarity and scientific strength, and we are looking forward to publishing the manuscript in PlosOne.

Sincerely,

Prof. Dr. Peter Breunig
