# Supplementary material for: Cover crops support the climate change mitigation potential of agroecosystems
Source: PLoS One. 2024 May 8;19(5):e0302139. doi: 10.1371/journal.pone.0302139 (PMC11078372; doi:10.1371/journal.pone.0302139)
Supplement: Attachment — Submitted filename: Comments.docx [file pone.0302139.s005.docx]

**Comments**

This manuscript reports systematic literature review study in which research platforms "Web of Science" and "Google Scholar" were used to explore the literature. In general, this manuscript is specific to cover crops significantly exceed their climate costs. Author claimed this study is first to include land use effects based on the yield gain caused by cover crops. Authors concluded that, if all maize cropping acreage in the EU-27 were to include cover crops, GHG emissions from EU-27 agriculture could be reduced by **13.0%.** Based on this result, authors recommend that the Common Agricultural Policy (CAP) of the European Union should continue to accelerate the integration of cover crops into farming.

The specific comments are given below.

- Clear take home message in the abstract is required.
- Abstract does not provide sufficient information about the literature review study.
- The Introduction is well written but does not present the reader with sufficient information on the problem being addressed.
- What are the objectives of this literature review study?
- The methods are largely well written. Although details of the methods adopted for this study provided in separate file, same is required to include in the methodology section.
- The Statistical Analysis is pretty much absent.
- The paper has no Conclusion and just kind of ends as per specific Journal style, instead author provided policy recommendations.
- Citations are complete and up to date.
- Overall, this paper needs a revision as per suggestions provided in the “Comment box” of edited paper as well as provided below.

**Line wise corrections suggested below**

**Line 45**

Instead “as well as lowerrequired fertilizers, pesticides, and water”

write “reduced agricultural”

**Line 58**

As an additional climate benefit, cover crops remove carbon from the

atmosphere during their growth while their residues and rhizodeposits are transformed to

stable soil organic matter…..**by retaining soil moisture.**

**Line 88**

(Table ). Table number is not mentioned.

Line 91

No references

**Line 178**

𝑅𝑒𝑑𝑢𝑐𝑒𝑑 𝑁2𝑂 − 𝑁 =

𝐸𝐹5 ∗ 𝑅𝑒𝑑𝑢𝑐𝑒𝑑 𝑁𝐿𝑒𝑎𝑐ℎ𝑖𝑛𝑔 ∗ 273

1000…

Supporting references?

Line 183&184

One by Kaye and Quemada [21] and another by Carrer et al. [14].

Delete this sentence write only reference

**Line 233 & 234**

Production emissions for seed production, dispatch and

additional machinery costs

Not understand what subtitle author want to represent

**Line 253 (253)**

How relative yield gain and absolute yield gain estimated?explain in methods section.

**Line 284**

Delete “found in” and insert sentence “reported by”

**Line 348**

Mention year ----IPCC (Year or reference number).

**Line 542**

Conclusion point is missing as per Journals format.
