# Supplementary material for: Cover crops support the climate change mitigation potential of agroecosystems
Source: PLoS One. 2024 May 8;19(5):e0302139. doi: 10.1371/journal.pone.0302139 (PMC11078372; doi:10.1371/journal.pone.0302139)
Supplement: Peer review history

# Peer review: Decision Letter 0

**DOI:** 10.1371/journal.pone.0302139.r001

**Authors:** Abhay Omprakash Shirale

17 Jan 2024

PONE-D-23-39906Cover crops support the climate change mitigation potential of agroecosystemsPLOS ONE

Dear Dr. Breunig,

Thank you for submitting your manuscript to PLOS ONE. After careful consideration, we feel that it has merit but does not fully meet PLOS ONE’s publication criteria as it currently stands. Therefore, we invite you to submit a revised version of the manuscript that addresses the points raised during the review process.

Please submit your revised manuscript by Mar 02 2024 11:59PM. If you will need more time than this to complete your revisions, please reply to this message or contact the journal office at plosone@plos.org. When you're ready to submit your revision, log on to https://www.editorialmanager.com/pone/ and select the 'Submissions Needing Revision' folder to locate your manuscript file.

Please include the following items when submitting your revised manuscript:

- A rebuttal letter that responds to each point raised by the academic editor and reviewer(s). You should upload this letter as a separate file labeled 'Response to Reviewers'.
- A marked-up copy of your manuscript that highlights changes made to the original version. You should upload this as a separate file labeled 'Revised Manuscript with Track Changes'.
- An unmarked version of your revised paper without tracked changes. You should upload this as a separate file labeled 'Manuscript'.

If you would like to make changes to your financial disclosure, please include your updated statement in your cover letter. Guidelines for resubmitting your figure files are available below the reviewer comments at the end of this letter.

If applicable, we recommend that you deposit your laboratory protocols in protocols.io to enhance the reproducibility of your results. Protocols.io assigns your protocol its own identifier (DOI) so that it can be cited independently in the future. For instructions see: https://journals.plos.org/plosone/s/submission-guidelines#loc-laboratory-protocols. Additionally, PLOS ONE offers an option for publishing peer-reviewed Lab Protocol articles, which describe protocols hosted on protocols.io. Read more information on sharing protocols at https://plos.org/protocols?utm_medium=editorial-email&utm_source=authorletters&utm_campaign=protocols.

We look forward to receiving your revised manuscript.

Kind regards,

Abhay Omprakash Shirale, PhD

Academic Editor

PLOS ONE

Journal Requirements:

1. When submitting your revision, we need you to address these additional requirements.

Please ensure that your manuscript meets PLOS ONE's style requirements, including those for file naming. The PLOS ONE style templates can be found at 

https://journals.plos.org/plosone/s/file?id=wjVg/PLOSOne_formatting_sample_main_body.pdf and 

https://journals.plos.org/plosone/s/file?id=ba62/PLOSOne_formatting_sample_title_authors_affiliations.pdf

2. Did you know that depositing data in a repository is associated with up to a 25% citation advantage (https://doi.org/10.1371/journal.pone.0230416)? If you’ve not already done so, consider depositing your raw data in a repository to ensure your work is read, appreciated and cited by the largest possible audience. You’ll also earn an Accessible Data icon on your published paper if you deposit your data in any participating repository (https://plos.org/open-science/open-data/#accessible-data).

3. Thank you for stating the following financial disclosure: 

The research was funded by the German Federal Ministry of Education and Research within the Project "CATCHY", project number: 031B1060C

Please state what role the funders took in the study.  If the funders had no role, please state: "The funders had no role in study design, data collection and analysis, decision to publish, or preparation of the manuscript." 

If this statement is not correct you must amend it as needed. 

Please include this amended Role of Funder statement in your cover letter; we will change the online submission form on your behalf.

4. Please include captions for your Supporting Information files at the end of your manuscript, and update any in-text citations to match accordingly. Please see our Supporting Information guidelines for more information: http://journals.plos.org/plosone/s/supporting-information. 

5. Please review your reference list to ensure that it is complete and correct. If you have cited papers that have been retracted, please include the rationale for doing so in the manuscript text, or remove these references and replace them with relevant current references. Any changes to the reference list should be mentioned in the rebuttal letter that accompanies your revised manuscript. If you need to cite a retracted article, indicate the article’s retracted status in the References list and also include a citation and full reference for the retraction notice.

[Note: HTML markup is below. Please do not edit.]

Reviewers' comments:

Reviewer's Responses to Questions

**Comments to the Author**

1. Is the manuscript technically sound, and do the data support the conclusions?

The manuscript must describe a technically sound piece of scientific research with data that supports the conclusions. Experiments must have been conducted rigorously, with appropriate controls, replication, and sample sizes. The conclusions must be drawn appropriately based on the data presented. 

Reviewer #1: Yes

Reviewer #2: Partly

**********

2. Has the statistical analysis been performed appropriately and rigorously? 

Reviewer #1: Yes

Reviewer #2: N/A

**********

3. Have the authors made all data underlying the findings in their manuscript fully available?

The PLOS Data policy requires authors to make all data underlying the findings described in their manuscript fully available without restriction, with rare exception (please refer to the Data Availability Statement in the manuscript PDF file). The data should be provided as part of the manuscript or its supporting information, or deposited to a public repository. For example, in addition to summary statistics, the data points behind means, medians and variance measures should be available. If there are restrictions on publicly sharing data—e.g. participant privacy or use of data from a third party—those must be specified.

Reviewer #1: Yes

Reviewer #2: Yes

**********

4. Is the manuscript presented in an intelligible fashion and written in standard English?

PLOS ONE does not copyedit accepted manuscripts, so the language in submitted articles must be clear, correct, and unambiguous. Any typographical or grammatical errors should be corrected at revision, so please note any specific errors here.

Reviewer #1: Yes

Reviewer #2: Yes

**********

5. Review Comments to the Author

Please use the space provided to explain your answers to the questions above. You may also include additional comments for the author, including concerns about dual publication, research ethics, or publication ethics. (Please upload your review as an attachment if it exceeds 20,000 characters)

Reviewer #1: Of course the study has lot of importance under current problematic agricultural situations to sustain both food production as well as soil health. The study findings are interesting. All the sections of the manuscript well defined and clearly written. Results with all data and discussion parts are described in detail giving appropriate justifications. However, in the manuscript, I found few minor corrections/modification, highlighted with yellow colors and comments within the manuscript itself for improvement. Further, many of grammatical language errors were found within the manuscript and need to be improve.

Reviewer #2: Comments

This manuscript reports systematic literature review study in which research platforms "Web of Science" and "Google Scholar" were used to explore the literature. In general, this manuscript is specific to cover crops significantly exceed their climate costs. Author claimed this study is first to include land use effects based on the yield gain caused by cover crops. Authors concluded that, if all maize cropping acreage in the EU-27 were to include cover crops, GHG emissions from EU-27 agriculture could be reduced by 13.0%. Based on this result, authors recommend that the Common Agricultural Policy (CAP) of the European Union should continue to accelerate the integration of cover crops into farming.

The specific comments are given below.

Clear take home message in the abstract is required.

Abstract does not provide sufficient information about the literature review study.

The Introduction is well written but does not present the reader with sufficient information on the problem being addressed.

What are the objectives of this literature review study?

The methods are largely well written. Although details of the methods adopted for this study provided in separate file, same is required to include in the methodology section.

The Statistical Analysis is pretty much absent.

The paper has no Conclusion and just kind of ends as per specific Journal style, instead author provided policy recommendations.

Citations are complete and up to date.

Overall, this paper needs a revision as per suggestions provided in the “Comment box” of edited paper as well as provided below.

 

Line wise corrections suggested below

Line 45

Instead “as well as lower required fertilizers, pesticides, and water”

write “reduced agricultural”

Line 58

As an additional climate benefit, cover crops remove carbon from the

atmosphere during their growth while their residues and rhizodeposits are transformed to

stable soil organic matter…..by retaining soil moisture.

Line 88

(Table ). Table number is not mentioned.

Line 91

No references

Line 178

 2 − =

5 ∗ ℎ ∗ 273

1000…

Supporting references?

Line 183&184

One by Kaye and Quemada [21] and another by Carrer et al. [14].

Delete this sentence write only reference

Line 233 & 234

Production emissions for seed production, dispatch and

additional machinery costs

Not understand what subtitle author want to represent

Line 253 (253)

How relative yield gain and absolute yield gain estimated?explain in methods section.

Line 284

Delete “found in” and insert sentence “reported by”

Line 348

Mention year ----IPCC (Year or reference number).

Line 542

Conclusion point is missing as per Journals format.

**********

6. PLOS authors have the option to publish the peer review history of their article (what does this mean?). If published, this will include your full peer review and any attached files.

If you choose “no”, your identity will remain anonymous but your review may still be made public.

**Do you want your identity to be public for this peer review?** For information about this choice, including consent withdrawal, please see our Privacy Policy.

Reviewer #1: **Yes: **Dr Vasudev Meena, ICAR-Directorate of Rapeseed-Mustard Research, Bharatpur, India

Reviewer #2: **Yes: **Dr. S.D. Jadhao, Professor, Department of Soil Science, Dr. PDKV, Akola

**********

[NOTE: If reviewer comments were submitted as an attachment file, they will be attached to this email and accessible via the submission site. Please log into your account, locate the manuscript record, and check for the action link "View Attachments". If this link does not appear, there are no attachment files.]

While revising your submission, please upload your figure files to the Preflight Analysis and Conversion Engine (PACE) digital diagnostic tool, https://pacev2.apexcovantage.com/. PACE helps ensure that figures meet PLOS requirements. To use PACE, you must first register as a user. Registration is free. Then, login and navigate to the UPLOAD tab, where you will find detailed instructions on how to use the tool. If you encounter any issues or have any questions when using PACE, please email PLOS at figures@plos.org. Please note that Supporting Information files do not need this step.

Attachment Submitted filename: PONE-D-23-39906_reviewer.pdf

Attachment Submitted filename: MS PLOS ONE.pdf

Attachment Submitted filename: Comments.docx

# Author comment: Author response to Decision Letter 0

**DOI:** 10.1371/journal.pone.0302139.r002

5 Mar 2024

Thank you for your supportive and insightful review of our manuscript “Cover crops support the climate change mitigation potential of agroeco- systems”. In the "Response to Reviewers" file we respond to each point raised by the academic editor and reviewers.

Attachment Submitted filename: Response to Reviewers.pdf

# Peer review: Decision Letter 1

**DOI:** 10.1371/journal.pone.0302139.r003

**Authors:** Abhay Omprakash Shirale

28 Mar 2024

Cover crops support the climate change mitigation potential of agroecosystems

PONE-D-23-39906R1

Dear Dr. Peter Breunig,

We’re pleased to inform you that your manuscript has been judged scientifically suitable for publication and will be formally accepted for publication once it meets all outstanding technical requirements.

Within one week, you’ll receive an e-mail detailing the required amendments. When these have been addressed, you’ll receive a formal acceptance letter and your manuscript will be scheduled for publication.

An invoice will be generated when your article is formally accepted. Please note, if your institution has a publishing partnership with PLOS and your article meets the relevant criteria, all or part of your publication costs will be covered. Please make sure your user information is up-to-date by logging into Editorial Manager at Editorial Manager® and clicking the ‘Update My Information' link at the top of the page. If you have any questions relating to publication charges, please contact our Author Billing department directly at authorbilling@plos.org.

If your institution or institutions have a press office, please notify them about your upcoming paper to help maximize its impact. If they’ll be preparing press materials, please inform our press team as soon as possible -- no later than 48 hours after receiving the formal acceptance. Your manuscript will remain under strict press embargo until 2 pm Eastern Time on the date of publication. For more information, please contact onepress@plos.org.

Kind regards,

Abhay Omprakash Shirale, PhD

Academic Editor

PLOS ONE

Reviewers' comments:

Reviewer's Responses to Questions

**Comments to the Author**

1. If the authors have adequately addressed your comments raised in a previous round of review and you feel that this manuscript is now acceptable for publication, you may indicate that here to bypass the “Comments to the Author” section, enter your conflict of interest statement in the “Confidential to Editor” section, and submit your "Accept" recommendation.

Reviewer #1: All comments have been addressed

Reviewer #2: (No Response)

**********

2. Is the manuscript technically sound, and do the data support the conclusions?

The manuscript must describe a technically sound piece of scientific research with data that supports the conclusions. Experiments must have been conducted rigorously, with appropriate controls, replication, and sample sizes. The conclusions must be drawn appropriately based on the data presented. 

Reviewer #1: Yes

Reviewer #2: Partly

**********

3. Has the statistical analysis been performed appropriately and rigorously? 

Reviewer #1: Yes

Reviewer #2: N/A

**********

4. Have the authors made all data underlying the findings in their manuscript fully available?

The PLOS Data policy requires authors to make all data underlying the findings described in their manuscript fully available without restriction, with rare exception (please refer to the Data Availability Statement in the manuscript PDF file). The data should be provided as part of the manuscript or its supporting information, or deposited to a public repository. For example, in addition to summary statistics, the data points behind means, medians and variance measures should be available. If there are restrictions on publicly sharing data—e.g. participant privacy or use of data from a third party—those must be specified.

Reviewer #1: Yes

Reviewer #2: Yes

**********

5. Is the manuscript presented in an intelligible fashion and written in standard English?

PLOS ONE does not copyedit accepted manuscripts, so the language in submitted articles must be clear, correct, and unambiguous. Any typographical or grammatical errors should be corrected at revision, so please note any specific errors here.

Reviewer #1: Yes

Reviewer #2: Yes

**********

6. Review Comments to the Author

Please use the space provided to explain your answers to the questions above. You may also include additional comments for the author, including concerns about dual publication, research ethics, or publication ethics. (Please upload your review as an attachment if it exceeds 20,000 characters)

Reviewer #1: All the raised comments or modifications incorporated and addressed well. However, in the reference section, the journals name not followed in same pattern, either write their abbreviation or elaboration.

Reviewer #2: Author addressed most of the comments of reviewer. Overall, this paper needs to refine considering minor revision as suggested. Finally after addressing all comments, handling editor to take decision on acceptance of MS for PLOS one. From my side paper accepted as author has been addressed most of the comments given during review process.

**********

7. PLOS authors have the option to publish the peer review history of their article (what does this mean?). If published, this will include your full peer review and any attached files.

If you choose “no”, your identity will remain anonymous but your review may still be made public.

**Do you want your identity to be public for this peer review?** For information about this choice, including consent withdrawal, please see our Privacy Policy.

Reviewer #1: **Yes: **Vasudev Meena, Crop Production Unit, ICAR-Directorate of Rapeseed-Mustard Research, Bharatpur, India- 321 303

Reviewer #2: **Yes: **Dr. Shyam Dalsing Jadhao

**********
